# Rbm24/Notch1 signaling regulates adult neurogenesis in the subventricular zone and mediates Parkinson-associated olfactory dysfunction

**DOI:** 10.7150/thno.96045

**Published:** 2024-08-01

**Authors:** Ke Wang, Xing-Yang Liu, Sui-Feng Liu, Xiao-Xia Wang, Yi-Hua Wei, Jun-Rong Zhu, Jing Liu, Xiu Qin Xu, Lei Wen

**Affiliations:** 1 Institute of Stem Cell and Regenerative Medicine, Women and Children's Hospital of Xiamen University, Fujian Provincial Key Laboratory of Neurodegenerative Disease and Aging Research, State Key Laboratory of Cellular Stress Biology, School of Medicine, Xiamen University, Xiamen, Fujian 361000, China.; 2 Center for Brain Sciences, Department of Traditional Chinese Medicine, The First Affiliated Hospital of Xiamen University, School of Medicine, Xiamen University, Xiamen, Fujian 361000, China.; 3 Zhongshan Hospital of Xiamen University, School of Medicine, Xiamen University, Xiamen, Fujian 361000, China.

**Keywords:** RNA binding motif protein 24, neurogenesis, subventricular zone, olfactory, Parkinson's disease

## Abstract

**Rationale:** Adult neurogenesis in the subventricular zone (SVZ) is essential for maintaining neural homeostasis, and its dysregulation contributes to anosmia and delayed tissue healing in neurological disorders, such as Parkinson's disease (PD). Despite intricate regulatory networks identified in SVZ neurogenesis, the molecular mechanisms dynamically maintaining neural stem/progenitor cells (NSPCs) in response to physiological and pathological stimuli remain incompletely elucidated.

**Methods:** We generated an RNA binding motif protein 24 (Rbm24) knockout model to investigate its impact on adult neurogenesis in the SVZ, employing immunofluorescence, immunoblot, electrophysiology, RNA-sequencing, and *in vitro* experiments. Further investigations utilized a PD mouse model, along with genetic and pharmacological manipulations, to elucidate Rbm24 involvement in PD pathology.

**Results:** Rbm24, a multifaceted post-transcriptional regulator of cellular homeostasis, exhibited broad expression in the SVZ from development to aging. Deletion of Rbm24 significantly impaired NSPC proliferation in the adult SVZ, ultimately resulting in collapsed neurogenesis in the olfactory bulb. Notably, Rbm24 played a specific role in maintaining Notch1 mRNA stability in adult NSPCs. The Rbm24/Notch1 signaling axis was significantly downregulated in the SVZ of PD mice. Remarkably, overexpression of Rbm24 rescued disruption of adult neurogenesis and olfactory dysfunction in PD mice, and these effects were hindered by DAPT, a potent inhibitor of Notch1.

**Conclusions:** Our findings highlight the critical role of the Rbm24/Notch1 signaling axis in regulating adult SVZ neurogenesis under physiological and pathological circumstances. This provides valuable insights into the dynamic regulation of NSPC homeostasis and offers a potential targeted intervention for PD and related neurological disorders.

## Introduction

Adult neurogenesis is a sustained and conserved process in the mammalian brains throughout life, impacting nervous system plasticity and acting as a crucial role in the pathogenesis of neurodegenerative disorders 1-4. Two specialized neurogenic niches exist in the adult brain: the subventricular zone (SVZ) of lateral ventricles (LV) and the subgranular zone (SGZ) of dentate gyrus (DG). Neural stem/progenitor cells (NSPCs) continuously undergo updating and iteration in these actively mitotic regions 1-4. Originating from the SVZ, the largest neurogenic area, adult-born neurons migrate and integrate into the existing neuronal microcircuits, particularly in the olfactory bulb (OB) or other afflicted regions, which is critical for the olfaction and tissue healing 5-7. Our previous research and relevant reports have shown that defective SVZ neurogenesis impedes the neural regeneration in the OB, leading to olfactory dysfunction across various mouse models of Parkinson's disease (PD) 8-10. Olfactory impairment, prevalent in 90% of PD patients, serves as an early diagnostic indicator of the disease and predicts a phenotype with worse posterior movement function 11-13. Mounting evidence supports the idea that reawakening neurogenic niches to rejuvenate the neural microenvironment is a promising therapeutic strategy for PD and related neurological diseases 9, 14-16. Although SVZ neurogenesis is known to respond to the crosstalk of extrinsic and intrinsic factors, involving RNA binding proteins (RBPs) 1, 17-20, the exact molecular mechanisms remain largely unknown.

RBPs play a critical role in the post-transcriptional regulation of targeted genes in physiology and pathology, with some identified as necessary for neurogenesis 19, 21, 22. Among them, RNA binding motif protein 24 (Rbm24) emerges as an evolutionarily conserved RBP. Our previous studies have demonstrated that Rbm24 regulates heart development, skeletal muscle regeneration and inner-ear recovery in a tissue-specific manner, establishing it as a key transcriptional regulator for cellular homeostasis and tissue development 23-29. Furthermore, Rbm24 has been implicated in directly regulating cell proliferation and tumor growth in several cancers 30-33. Despite the accumulating evidence pointing to the role of Rbm24 in cellular development, its functions in the regulation of NSPC dynamics in the SVZ under steady state, as well as its involvement in neuropathology, have not been well studied.

In the present study, we generated tamoxifen (TAM)-inducible Rbm24 knockout mice to elucidate its roles in adult SVZ neurogenesis under physiological and pathological conditions. Through RNA-sequencing (RNA-Seq) analysis, we identified a novel transcriptional regulatory role of Rbm24 in adult SVZ neurogenesis, with Notch1 mRNA identified as a direct target of Rbm24. Our findings further underscore the crucial role of the Rbm24/Notch1 signaling axis in adult SVZ neurogenesis and olfaction in PD mice, highlighting its potential as a therapeutic target for PD.

## Methods

### Animals

The UBC^CreERT2^ mice were obtained from Shanghai Model Organisms Center, China. The generating strategy of Rbm24^loxP/loxP^ mice was described previously 23. Heterozygous offspring of the overexpressed human A53T mutant mice (line G2-3, B6.Cg-Tg [Prnp-SNCA*A53T] 23 Mkle/J) were procured from Jackson Laboratories, USA. All animals were housed under standard conditions in a 12-h light/dark cycle, with free access to food and water. An approximately equal number of male and female mice were included in the experiments. All experiments and animal care were performed in compliance with the guidelines of the Xiamen University Animal Care and Use Committee (approval ID: XMULAC20170241).

### Primary culture of SVZ-derived adult NSPCs

Neurospheres formed by adult SVZ NSPCs were cultured based on previous study 34. Two-month-old mice were first injected with TAM (MCE, USA) and then euthanized 10 days later to expose the cerebrum. The cerebellum, OB, and cerebral cortex were removed, and SVZ tissues surrounding the LV were isolated under a stereomicroscope. The SVZ tissues were digested using 0.025% trypsin-EDTA and 0.7 mg/mL hyaluronidase at 37℃ for 20 min, with ovomucoid used to halt the digestion process. After the tissues were dispersed into single cell suspensions, cells were seeded into 24-well plates and cultured in neural stem cell (NSC) medium supplemented with fibroblast growth factor 2 and epidermal growth factor (both at 20 ng/mL, Thermo Fisher Scientific, USA). For *in-vitro* proliferation assays, 10 μM 5-bromo-2'-deoxyuridine (BrdU, Aladdin Biochemical Technology, China) was added to the cultured NSPCs. Then, a total of five animals were used for each condition. Neurosphere quantification was based on the analysis of two sections per glass coverslip per animal. The number and diameter of neurosphere were measured using ImageJ software with the Cell Counts plugins. Results were expressed as the averaged number or diameter of neurosphere per section.

### Intraperitoneal injection

Two-month-old Rbm24^loxP/loxP^ (Control, CTL) and UBC^CreERT2^; Rbm24^loxP/loxP^ (Inducible knockout, UKO) mice were intraperitoneally (i.p.) injected with TAM, dissolved in corn oil at a concentration of 20 mg/mL, at a daily dose of 0.1 g/kg body weight for 5 consecutive days. Mice were sacrificed at various time points following TAM induction for different assays: 10 days for knockout efficiency analysis of Rbm24, 11 days for active NSPC proliferation assays in the SVZ, 47 days for quiescent NSCs assays in the SVZ, 17 days for migrating cells assays in the rostral migratory stream (RMS), and 45 days for adult-born neurons assays in the OB.

For the Notch1 inhibiting assays, 7-month-old mice were administered i.p. with the γ-secretase inhibitor N-[N-(3,5-difluorophenacetyl)-l-alanyl]-Sphenylglycinet-butylester (DAPT, Sigma, USA) at a daily dose of 10 mg/kg body weight, following a protocol established in a previous study 35. To assess the effects of DAPT on active NSPC proliferation, DAPT was administered once daily for 7 days, starting from the 14th day post lentiviral injection. For evaluating the long-term impact of DAPT on olfactory behaviors, DAPT was administered once daily for 14 days, commencing from the 14th day post lentiviral injection.

### Stereotactic virus injections

To label adult-born neurons migrating from the SVZ to the OB, adeno-associated virus 1 (AAV1) encoding enhanced green fluorescent protein (EGFP) under the control of the cytomegalovirus (CMV) promoter was purchased from Han Heng biotechnology, China. As previously described 8, 500 nL AAV1 was stereotactically bilaterally infused into the RMS of 2-month-old mice with coordinates relative to Bregma: AP, -3.3 mm; ML, ±0.8 mm; DV, -2.9 mm. Mice were sacrificed for examination 28 days post AAV1 injection.

For Rbm24 overexpression in the SVZ, lentivirus (2×10^8^ TU/mL) encoding Rbm24 and green fluorescent protein (GFP) under the control of the CMV promoter was purchased from FENGHUI biotechnology, China. Following a previously established protocol 36, 10 μL lentivirus was stereotactically bilaterally infused into the SVZ of 7-month-old mice (coordinates relative to Bregma: AP, -0.1 mm; ML, ±1.38 mm; DV, -2.6 mm). For overexpression efficiency assays of Rbm24, mice were sacrificed 14 days after lentiviral infection. For active NSPC proliferation assays in the SVZ, mice were sacrificed 15 days after lentiviral infection. For adult-born neurons assays in the OB, mice were sacrificed 48 days after lentiviral infection.

### *In-vivo* proliferation assays

For the *in-vivo* proliferation assays, 2-month-old (Rbm24 deletion condition) and 7-month-old (PD with Rbm24 overexpression condition) mice were administered i.p. with BrdU at a dose of 100 mg/kg body weight based on previous studies 8, 36. To label actively proliferating cells in the SVZ, BrdU was injected into the mice three times at 2 h intervals, and these mice were sacrificed 24 h post BrdU injection. To label quiescent cells in the SVZ, BrdU was injected into the mice once daily for 7 days and these mice were sacrificed 30 days post BrdU injection. To label migrating cells in the RMS, BrdU was injected into the mice three times at 2 h intervals and these mice were sacrificed 7 days post BrdU injection. To label adult-born cells in the OB, BrdU was injected into the mice once daily for 5 days and these mice were sacrificed 30 days post BrdU injection.

### Immunohistochemical and immunocytochemical staining

Immunohistochemical staining was performed as previously established protocols 8, 37. Mice were euthanized under deep anesthesia and perfused with 0.01 M phosphate-buffered saline (PBS, MeilunBio, China) and 4% paraformaldehyde (PFA, MeilunBio, China). Slices (20 μm thickness) from the SVZ, RMS, and OB were collected and incubated overnight at 4 ℃ with primary antibodies: Rbm24 (1:200, ab94567, Abcam, UK), GFAP (1:500, 3670S, Cell Signaling Technology, USA), DCX (1:300, sc271390, Santa Cruz, USA), BrdU (1:250, ab6326, Abcam, UK), Caspase-3 (1:100, T40044, Abmart, China), NeuN (1:500, ab177487, Abcam, UK), TH (1:200, ab1542, Sigma, USA), CalR (1:100, sc365956, Santa Cruz, USA), CalB (1:200, 13176S, Cell Signaling Technology, USA), Notch1 (1:100, 3447S, Cell Signaling Technology, USA), and GFP (1:300, ab290, Abcam, UK).

For immunocytochemical staining in NSPC cultures, cells were fixed with 4% PFA, permeabilized with Triton X-100, and blocked with 5% bovine serum albumin (BSA, Beyotime Biotechnology, China). The cultures were incubated with primary antibodies: Rbm24 (1:200, ab94567, Abcam, UK), GFAP (1:500, 3670S, Cell Signaling Technology, USA), Sox2 (1:200, sc365823, Santa Cruz, USA), and BrdU (1:250, ab6326, Abcam, UK).

Both tissue slices and cultures were incubated with appropriate secondary antibodies: Alexa Fluor 488 Goat anti-Mouse IgG (1:1000, A-11001, Thermo Fisher Scientific, USA), Alexa Fluor 488 Goat anti-Rat IgG (1:1000, A-11006, Thermo Fisher Scientific, USA), Alexa Fluor 488 Goat anti-Rabbit IgG (1:1000, A-11008, Thermo Fisher Scientific, USA), Alexa Fluor 594 Goat anti-Mouse IgG (1:1000, A-11005, Thermo Fisher Scientific, USA), Alexa Fluor 594 Goat anti-Rat IgG (1:1000, A-11007, Thermo Fisher Scientific, USA), Alexa Fluor 594 Goat anti-Rabbit IgG (1:1000, A-11012, Thermo Fisher Scientific, USA), Alexa Fluor 647 Goat anti-Rat IgG (1:1000, A-21247, Thermo Fisher Scientific, USA), and DAPI (Beyotime Biotechnology, China).

Images were captured using a laser-scanning confocal microscope (Nikon, Japan). Quantitative analyses were performed on one or two sections per tissue slide or glass coverslip per animal, with three or four animals included per group. Target cells were counted using ImageJ software with the Cell Counts plugins. Results were expressed as the number of cells per section.

### TUNEL assay

Based on the manufacturer's instructions, slices (20 μm thickness) from the OB were subjected to TUNEL assay by using a TUNEL apoptosis kit (Beyotime Biotechnology, China). Images were captured using a laser-scanning confocal microscope (Nikon, Japan). Quantitative analyses were performed on two or three sections per tissue slide per animal, with four animals included per group. Apoptotic cells were counted using ImageJ software with the Cell Counts plugins. Results were expressed as the number of cells per section.

### Quantitative PCR and RT-PCR

As previously described 27, 37, RNA extraction was performed using the Trizol^®^ reagent (Thermo Fisher Scientific, USA) according to the manufacturer's instructions. 1 µg total RNA was used for cDNA synthesis with the All-in-one Supermix (Transgen, China). PCR was amplified for 25-30 cycles, and the resulting products were detected using high-resolution agarose gel electrophoresis. Quantitative PCR (q-PCR) was performed using a STEP ONE instrument with a reaction volume of 20 μL containing 25 ng templates. The primers were listed in Table S1.

### Immunoblotting

Following established protocols 8, 30, 38, SVZ tissues surrounding the LV were isolated, and total proteins were extracted using radioimmunoprecipitation assay (RIPA) lysis buffer (50 mM Tris-HCl pH 7.4, 150 mM NaCl, 1% Triton X-100, 0.1% SDS, 1% Sodium deoxycholate, 0.5 mM PMSF) after exposing the cerebrum. Protein quantitation was performed with the BCA kit (Beyotime Biotechnology, China). Samples were subjected to electrophoresis and transferred to polyvinylidene fluoride membranes. After blocking with 5% BSA, membranes were incubated with primary antibodies against Rbm24 (1:3000, ab94567, Abcam, UK), Notch1 (1:1000, 3447S, Cell Signaling Technology, USA), NICD (1:1000, 4147, Cell Signaling Technology, USA) and GAPDH (1:10000, 60004-1-Ig, Proteintech, China). Appropriate secondary antibodies were applied: Goat anti-Mouse IgG HRP (1:10000, 31430, Thermo Fisher Scientific, USA), Goat anti-Rat IgG HRP (1:10000, 31470, Thermo Fisher Scientific, USA), Goat anti-Rabbit IgG HRP (1:10000, 31460, Thermo Fisher Scientific, USA). Immunoreactive bands were visualized with chemiluminescence reagents (Beyotime Biotechnology, China). For the analysis of immunoreactive bands, six animals were used for each condition. Quantitative analysis of bands was performed using densitometry with ImageJ software, and values were expressed relative to GAPDH.

### RNA-seq

SVZ sections from 2-month-old mice (n = 4 for CTL, n = 3 for UKO), collected at 10th day after TAM injection, underwent RNA extraction for RNA-seq analysis. The sequencing library was prepared following the manufacturer's protocol (NEBNext^®^ Ultra^™^ RNA Library Prep Kit for Illumina^®^; New England BioLabs, USA). RNA samples were sequenced to a depth of 40-60 million reads, with a 2×150 bp paired-end configuration on an Illumina system. Subsequently, reads were aligned to the reference mouse genome (GRCm38/mm10) using TopHat. Transcript assembly and expression abundance analysis were conducted with Cufflink. Differentially expressed genes (DEGs) were determined based on a screening criterion of log2 fold change (|log2FC|) > 1 and false discovery rate (FDR) < 0.05. DEGs were annotated using the cluster Profiler R package, including Gene Ontology (GO) functional analysis and Kyoto Encyclopedia of Genes and Genomes (KEGG) metabolic pathway analysis.

### RNA immunoprecipitation

As a previous study with minor modifications 25, SVZ sections from 2-month-old CTL mice were collected and mixed with cell lysate and magnetic beads before being placed into the tissue grinder. Subsequently, the samples were transferred into tubes and incubated with magnetic beads overnight at 4 ℃. After incubation, the RNA pulled down by Rbm24 was eluted from the magnetic beads using Trizol, followed by the standard procedures for RNA extraction and assessment.

### Luciferase reporter assays

As described previously 39, the 3'UTR sequence of Notch1 gene was PCR amplified from purified mouse cortical genomic DNA and then cloned into psiCheck2 dual luciferase vector using T4 DNA ligase (Thermo Fisher Scientific, USA). The Rbm24 promoter-reporter plasmids were cloned into the pcDNA3.1 vector. Transfection of 293T cells was carried out using PEI transfection reagent (Sigma, USA) following the manufacturer's protocol. The transfected cells were collected and subjected to luciferase activity assays, assessed immediately using a Tecan Infinite 200 Pro One plate reader (Tecan Trading AG, CH).

### Electrophysiology

Based on previous studies with minor modifications 8, 37, 38, OB horizontal slices (350 μm) from 4-month-old (Rbm24 deletion condition) or 9-month-old (PD with Rbm24 overexpression condition) mice were prepared using a vibratome (Leica, DEU). Subsequently, these slices underwent ice-cold, oxygenated (95% O_2_, 5% CO_2_) incubation, in a solution containing (in mM, pH 7.2-7.4): N-methyl-D-glucamine (93), HCl (93), KCl (2.5), NaH_2_PO_4_ (1.2), NaHCO_3_ (30), HEPES (20), glucose (25), (+)-sodium L-ascorbate (5), thiourea (2), sodium pyruvate (3), CaCl_2_ (0.5), and MgSO_4_ (10). Following this, the slices were transferred to the oxygenated artificial cerebral spinal fluid containing (in mM, pH 7.2-7.4): NaCl (124), KCl (2.5), CaCl_2_ (2), NaH_2_PO_4_ (1.2), NaHCO_3_ (24), MgSO_4_ (2), glucose (12.5), and HEPES (5), and this solution was employed for subsequent recording. For action potential (AP) recording, neurons with GFP were located and recorded with pipettes (3-8 MΩ) filled with a specific internal solution under whole-cell current-clamp mode. The AP was evoked by a depolarizing 200-pA current step for 300 ms. The internal solution for AP recording included (in mM, pH 7.2-7.4): K-gluconate (125), NaCl (5), HEPES (10), EGTA (10), MgCl_2_ (2), and Na_2_-GTP (2). For spontaneous inhibitory postsynaptic current (sIPSC) recording, the mitral/tufted cells (M/Ts) were recorded with pipettes (3-8 MΩ) filled with specific internal solution under whole-cell voltage-clamp mode (holding at 0 mV). The internal solution for sIPSC recording included (in mM, pH 7.2-7.4): CsCH_3_SO_3_ (140), MgCl_2_ (2), TEA-Cl (5), HEPES (10), EGTA (1), Mg-ATP (2.5), and Na_2_-GTP (0.3). Data were filtered at 2 kHz, sampled at 10 kHz using an Axon MultiClamp 700B amplifier (Molecular Devices, USA), and digitized with pClamp 10 software (Molecular Devices, USA). Quantitative analysis of electrophysiological data was performed using Clampfit 10 software (Molecular Devices, USA).

### Buried pellet test

In line with previous study 8, 9-month-old mice, 60 days post lentiviral injection, underwent a 24-h period of food deprivation with access to water ad libitum. After habituation in the test cage, mice were introduced into a clear test cage with 3-cm-deep bedding and tasked with exploring an approximately 400-mg food pellet buried 1 cm beneath the bedding in a randomly selected corner. The latency to locate the food pellet was measured within a 300-s timeframe. A visual food pellet test was conducted as a control, where mice were subjected to find the food pellet on the surface of the bedding in the same test cage. The latency to locate the food pellet was recorded. To ensure consistency, mice were initially positioned at an equal distance from both the buried and visual food pellets. All behavioral experiments were conducted consistently within a set schedule between 9 am and 3 pm.

### Olfactory preference/avoidance test

Based on our previous report 8, 1 g peanut butter (PB) mixed with 50 µL of mineral oil was served as the attractive odor (PB solution), while 50 µL 2,4,5-trimethylthiazoline (TMT), a compound eliciting innate fear in rodents found in fox feces, diluted to 40% v/v in mineral oil, was used as the aversive odor (TMT solution). 60 days post lentiviral injection, 9-month-old mice were placed into an empty test cage for 5-min acclimation. Mineral oil (20 µL) was applied to filter paper in the cage as a control odor. For the odor preference or avoidance test, 20 µL of PB or TMT solution was applied to the filter paper, respectively. The mice were allowed to sniff the filter paper containing the odor solution freely for 5 min, and the time spent sniffing the filter paper was recorded. Sniffing was defined as the distance between the mouse's nose and the center of filter paper being within 1 cm. All behavioral experiments were conducted consistently within a set schedule between 9 am and 3 pm.

### Olfactory discrimination test

Based on our previous report 8, 9-month-old mice were kept in separate cages and deprived of water but had free access to food for 24 h prior to the experiment. During the training phase, each mouse was kept in a clean cage, where the mango odor was associated with a tasty drink and the almond odor was associated with a bitter drink. The mixture of distilled water and mango extract (Mgo), defined as “+”, was used as a reward, while the combination of almond extract (ALM) with 1% denatonium benzoate (DB) solution, defined as “-”, was used as a penalty. Then, in a clean cage, 10 µL of distilled water and Mgo mixture was added to a 35×10 mm sterile dish to acclimate the mice to the Mgo odor. The volume of Mgo was gradually increased to 1, 2.5, 4, 5.5, 7, and 8.5 µL, with each mouse allowed to explore the “+” solution for 2 min, with 30 s intervals between trials. Subsequently, 8.5 µL of ALM mixed with 1.5 µL of 1% DB solution was provided to associate the bitter taste with ALM, repeating the test four times. During the testing phase, the “+” and “-” solutions were placed in two dishes in a test cage and mixed in different proportions. One dish mainly held the “+” solution, and the other dish primarily held the “-” solution. For instance, a 60:40 ratio of the odor solution indicated a 60:40 compositional ratio of Mgo in distilled water to 1% DB of ALM in one dish, and the opposite ratio in the other dish. If a mouse chose only the “+” solution within 30 s, it was deemed a success. If a mouse chose the “-” solution or both solutions within 30 s, it was considered a failure. Mice that did not select either solution were excluded from the analysis. Each mouse underwent ten trials, and the percentage of success in olfactory discrimination was calculated. All behavioral experiments were conducted consistently between 9 am and 3 pm.

### Statistics

Statistical analysis of data was conducted with GraphPad Prism 9.4.1 software. All data were plotted as the mean ± standard error of the mean (SEM), with significance set at *P* < 0.05. For two-group comparison, unpaired *t* test or nonparametric Kolmogorov-Smirnov test was applied. For multiple comparison, two-way analysis of variance (ANOVA) was conducted, followed by the Tukey's test for *post hoc* analysis.

## Results

### Rbm24 is broadly expressed in NSPCs of the SVZ

**Figure 1:**
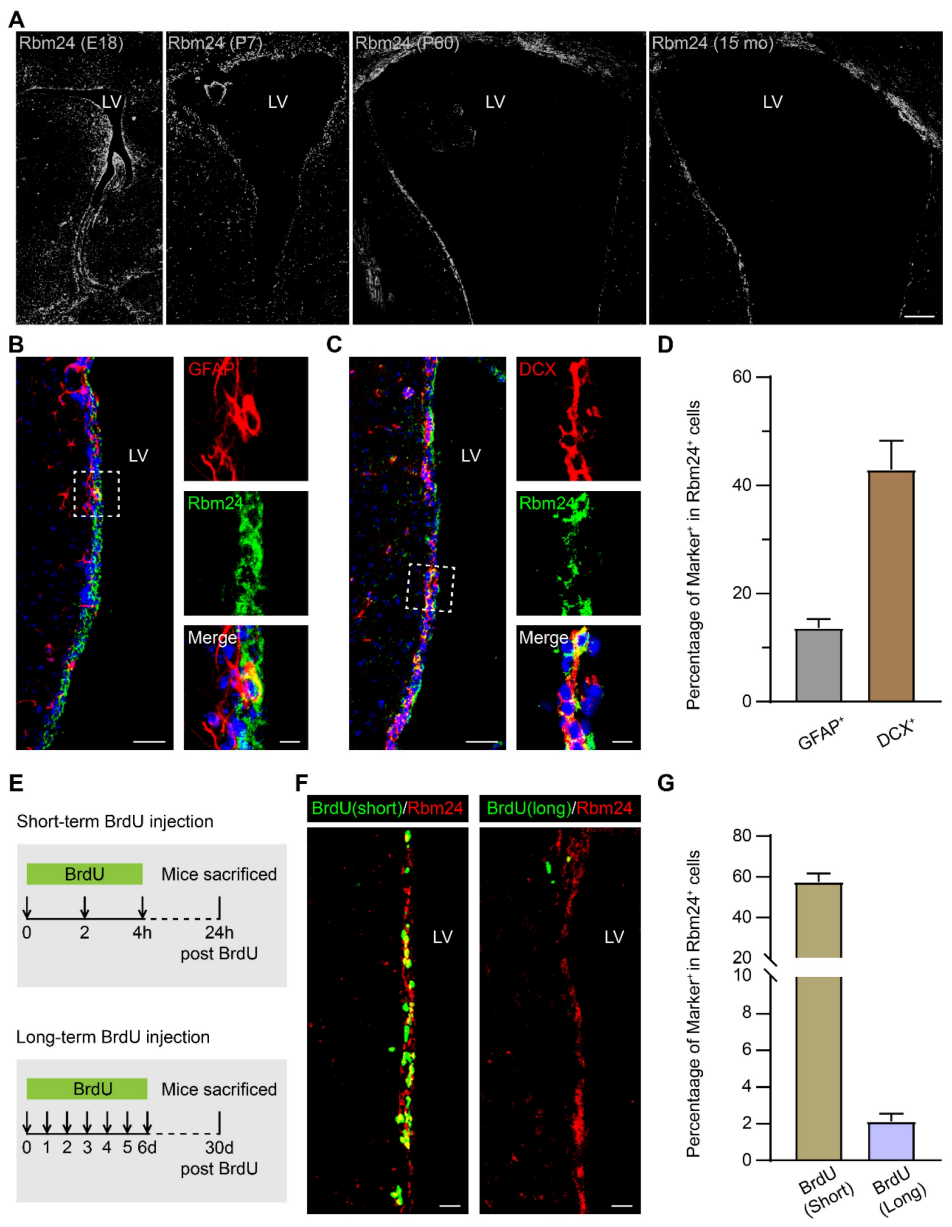
**Rbm24 is broadly expressed in the SVZ during the development and aging. (A)** Representative images of Rbm24 distribution in the SVZ from CTL mice at different developmental stages. Scale bar, 100 μm. **(B-C)** Representative images of GFAP/Rbm24 (**B**) and DCX/Rbm24 (**C**) co-staining in the SVZ from 2-month-old CTL mice. Scale bar, 50 μm, zoom scale bar, 10 μm. **(D)** Percentage of GFAP^+^ and DCX^+^ cells in Rbm24^+^ cells in the SVZ from 2-month-old CTL mice (n = 4 mice). **(E)** Timeline of short-term and long-term BrdU injection. **(F)** Representative images of Rbm24/BrdU (left panel indicated short-term, right panel indicated long-term) co-staining in the SVZ from 3-month-old CTL mice. Scale bar, 20 μm. **(G)** Percentage of BrdU^+^/Rbm24^+^ cells in Rbm24^+^ cells in the SVZ from 2-3-month-old CTL mice (n = 4 mice). LV: lateral ventricle; E18: embryonic 18; P7: postnatal 7; P60: postnatal 60; 15 mo: 15 months of old.

To determine the role of Rbm24 in the SVZ, we first examined its temporal expression pattern. Immunostaining analysis across various developmental stages, including embryonic (E18), post-natal (P7), adult (P60), and aged (15 months) mice, revealed a robust and widespread expression of Rbm24 in the ventricular zone and SVZ of the neocortex at diverse stages (Figure 1A). This pervasive expression suggests a potential involvement of Rbm24 in the SVZ neurogenesis across development.

We further explored the specific expression profile of Rbm24 along the SVZ-OB pathway in 2-month-old mice, utilizing cellular lineage markers to define cell types (Figure S1A). Rbm24 was highly expressed in the glial fibrillary acidic protein-positive (GFAP^+^) type-B NSCs and doublecortin-positive (DCX^+^) type-A neuroblasts in the SVZ (Figure 1B-D). These findings align with previous scRNA-seq data from the adult SVZ in mice 40. While type-A neuroblasts typically migrate from the SVZ into the OB along the RMS, Rbm24 expression was infrequent in the DCX^+^ type-A neuroblasts in both the RMS and OB (Figure S1B-C).

In addition, we evaluated the proliferation status of Rbm24^+^ cells through a BrdU incorporating assay (Figure 1E). Short-term BrdU immunostaining, labeling the actively proliferating NSPCs, exhibited co-staining with BrdU in the majority of Rbm24^+^ cells (Figure 1F-G). Long-term BrdU retention experiments, designed to identify the slowly cycling quiescent NSCs, revealed a smaller population of Rbm24^+^/BrdU^+^ colocalized cells in the SVZ (Figure 1F-G). In conclusion, Rbm24 is mainly expressed in the actively proliferating NSPCs in the SVZ, with a few involved in the quiescent NSCs in the SVZ, suggesting a potential function for Rbm24 in adult neurogenesis in the SVZ.

### Rbm24 deficiency affects adult neurogenesis in the SVZ

**Figure 2:**
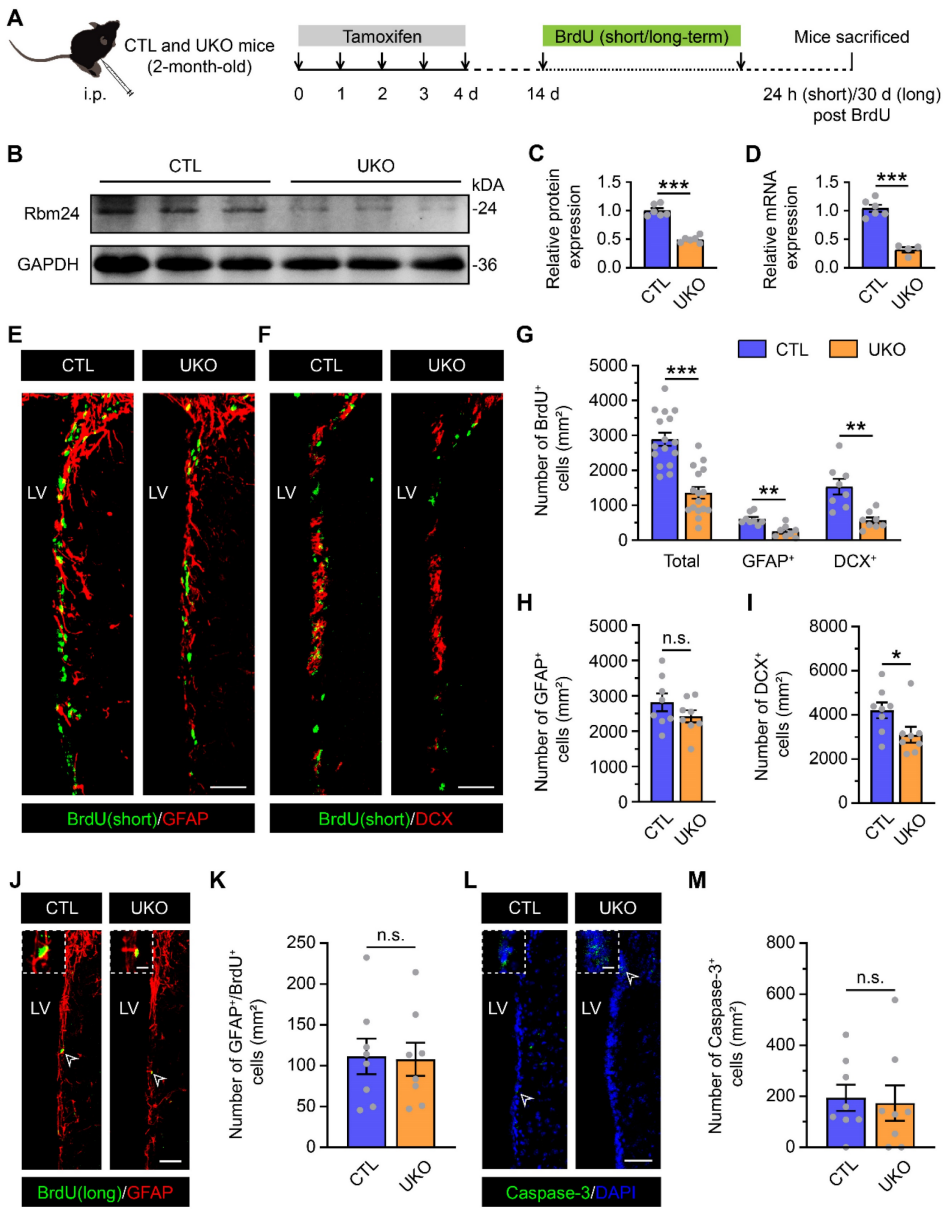
** Rbm24 deficiency alters the number of actively proliferative but not quiescent NSPCs in the adult SVZ. (A)** Timeline of tamoxifen and BrdU (short/long-term) administration in 2-month-old CTL and UKO mice. **(B-C)** Immunoblot (**B**) and quantification (**C**) of Rbm24 protein in the SVZ from 2-month-old CTL and UKO mice (n = 6 mice for each group). **(D)** Quantification of Rbm24 mRNA in the SVZ from 2-month-old CTL and UKO mice (n = 6 mice for each group). **(E-F)** Representative images of BrdU (short-term)/GFAP (**E**) and BrdU (short-term)/DCX (**F**) co-staining in the SVZ from 2-month-old CTL and UKO mice. Scale bar, 50 μm. **(G)** Quantification of BrdU^+^ (short-term), BrdU^+^ (short-term)/GFAP^+^, BrdU^+^ (short-term)/DCX^+^ cells in the SVZ from 2-month-old CTL and UKO mice (n = 4 mice for each group). **(H-I)** Quantification of GFAP^+^ (**H**), DCX^+^ (**I**) cells in the SVZ from 2-month-old CTL and UKO mice (n = 4 mice for each group). **(J-K)** Representative images (**J**) and quantification (**K**) of BrdU^+^ (long-term)/GFAP^+^ cells in the SVZ from 3-month-old CTL and UKO mice (n = 4 mice for each group). Scale bar, 50 μm, zoom scale bar, 10 μm. **(L-M)** Representative images (**L**) and quantification (**M**) of Caspase-3^+^ cells in the SVZ from 2-month-old CTL and UKO mice (n = 4 mice for each group). Scale bar, 50 μm, zoom scale bar, 10 μm. Data are presented as mean ± SEM. *p < 0.05; **p < 0.01; ***p < 0.001; n.s., not significant. i.p.: intraperitoneally; LV: lateral ventricle.

To dissect the potential roles of Rbm24 in SVZ neurogenesis, we generated a specific type of Rbm24 knockout mice, designated as UKO mice. These mice featured an inducible Rbm24 deletion through continuous TAM administration. The control counterparts, denoted as CTL mice, possessed Rbm24^loxP/loxP^ without CreERT2 (Figure 2A). Immunoblot and q-PCR analysis confirmed a significant deletion of Rbm24 in the SVZ of 2-month-old UKO mice following TAM injection (Figure 2B-D), further corroborated by Rbm24 immunostaining (Figure S2A-B).

Subsequently, we examined whether Rbm24 deficiency influenced adult neurogenesis in the SVZ. The SVZ of UKO mice exhibited fewer BrdU^+^ cells compared to CTL mice (Figure 2E-G). Immunostaining further revealed that GFAP^+^/BrdU^+^ proliferating type-B NSCs and DCX^+^/BrdU^+^ dividing type-A neuroblasts were significantly reduced in the SVZ of UKO mice relative to CTL mice (Figure 2E-G), indicating a substantial reduction in actively proliferating NSPCs. There were fewer DCX^+^ type-A neuroblasts in the SVZ of UKO mice compared to CTL mice, while the number of GFAP^+^ type-B NSCs remained unchanged (Figure 2H-I). This observation might be attributed to the fact that GFAP^+^ type-B cells, encompassing both quiescent and activated NSCs, with the latter accounting for only a small fraction. Rbm24 deficiency seems to selectively impact activated, but not quiescent NSCs in the physical state. This notion is supported by the long-term BrdU incorporation analysis (Figure 2J-K). Moreover, we explored whether the reduction in actively proliferating NSPCs resulted from increased apoptosis in the SVZ. However, the number of caspase-3^+^ cells in UKO mice was minimal and similar to that in CTL mice (Figure 2L-M). Consequently, these findings propose a crucial role for Rbm24 in the proliferation of activated NSPCs in the adult SVZ.

### Rbm24 deficiency impedes adult neurogenesis in the OB

**Figure 3:**
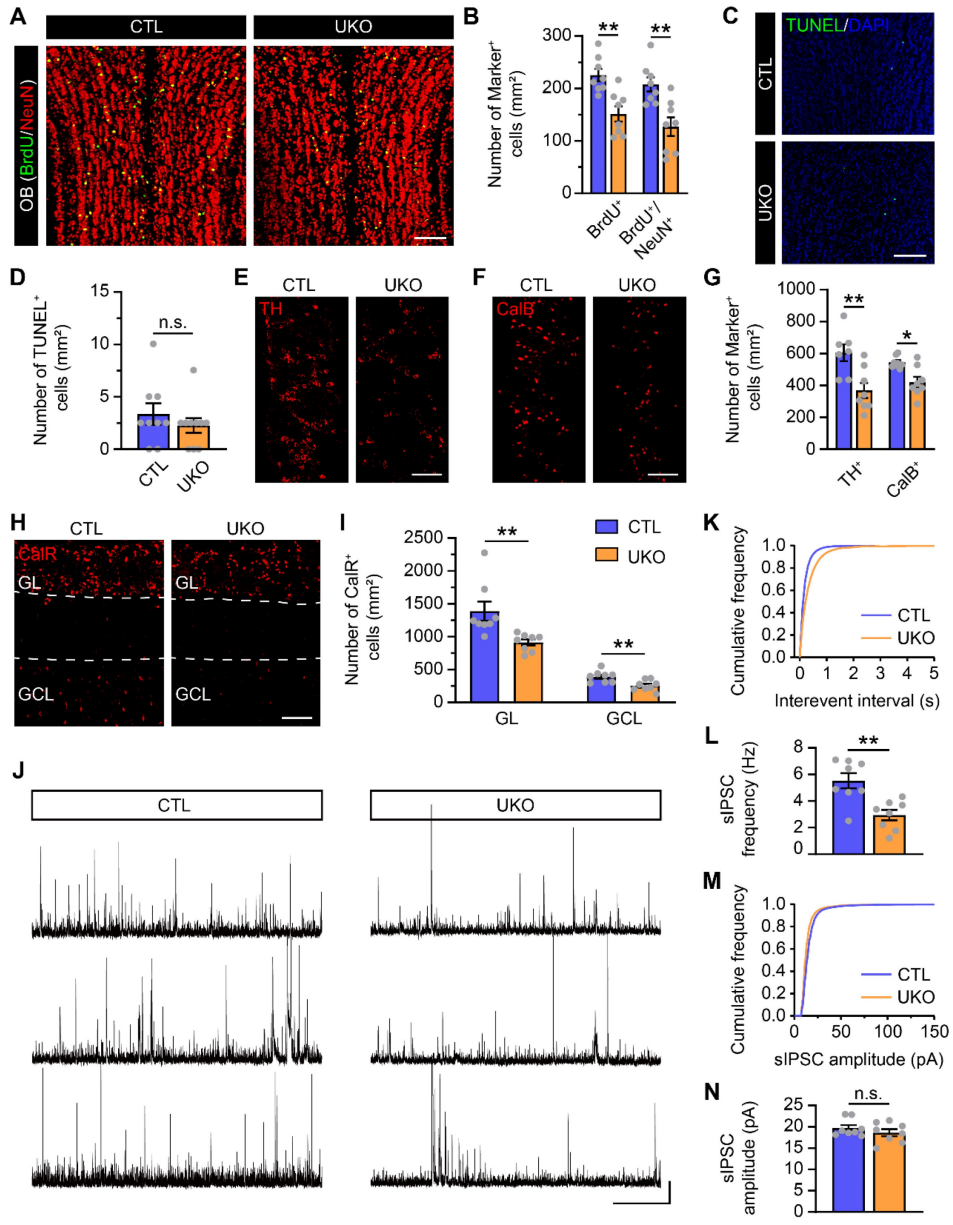
** Rbm24 deficiency impedes adult neurogenesis in the OB. (A)** Representative images of BrdU/NeuN co-staining in the OB from 3-month-old CTL and UKO mice. Scale bar, 100 μm. **(B)** Quantification of BrdU^+^ and BrdU^+^/NeuN^+^ cells in the OB from 3-month-old CTL and UKO mice (n = 4 mice for each group). **(C-D)** Representative images and quantification of TUNEL^+^ cells in the OB from 3-month-old CTL and UKO mice (n = 4 mice for each group). Scale bar, 100 μm. **(E-I)** Representative images and quantification of TH^+^ (**E, G**), CalB^+^ (**F, G**), CalR^+^ (**H, I**) cells in the OB from 4-month-old CTL and UKO mice 60 days after TAM administration (n = 4 mice for each group). Scale bar, 100 μm. **(J)** Representative traces of sIPSC of M/Ts in the OB from 4-month-old CTL and UKO mice. Scale bar, 40 pA and 20 s. **(K)** Cumulative curves of sIPSC interevent interval of M/Ts in the OB from 4-month-old CTL and UKO mice. **(L)** Quantification of sIPSC frequency of M/Ts in (**J**) (n = 4 mice for each group). **(M)** Cumulative curves of sIPSC amplitude of M/Ts in the OB from 4-month-old CTL and UKO mice. **(N)** Quantification of sIPSC amplitude of M/Ts in (**J**) (n = 4 mice for each group). Data are presented as mean ± SEM. *p < 0.05; **p < 0.01; n.s., not significant. OB: olfactory bulb; GL: glomerular layer; GCL: granule cell layer.

In the SVZ-OB pathway, neuroblasts undergo migration, maturation, and integration into the OB neural microcircuits. To further understand the potential consequences of Rbm24 deficiency on adult neurogenesis in the OB, we conducted additional evaluations. Initially, a decrease in the population of BrdU^+^ and BrdU^+^/NeuN^+^ cells, termed adult-born neurons, was observed in the OB of UKO mice, compared to CTL mice (Figure 3A-B). Moreover, we also examined whether the reduction of adult-born neurons results from an increased apoptosis in the OB. However, TUNEL analysis revealed a similar number of apoptotic cells in the OB of CTL and UKO mice (Figure 3C-D). Furthermore, the overall count of calretinin-positive (CalR^+^), calbindin-positive (CalB^+^), tyrosine hydroxylase-positive (TH^+^) neurons in the OB of UKO mice were similar to those in CTL mice 40 days post TAM injection (Figure S3A-F).

However, a reduction in all CalR^+^, CalB^+^, TH^+^ neurons became evident in the OB of UKO mice 60 days after the TAM administration (Figure 3E-I). This aligns with findings from electrophysiological assays, which revealed a diminished sIPSC frequency of M/Ts in the OB of UKO mice, while the amplitude remained unchanged (Figure 3J-N). Considering the reduction of adult-born neurons in the OB results from not only the decreased neurogenesis in the SVZ but also the aberrant migration of neuroblasts in the RMS, the BrdU-tracing experiments were conducted to detect the migration. In the SVZ-OB pathway, the percentages of BrdU^+^ cells in the SVZ, RMS and OB of UKO mice were similar to those in CTL mice (Figure S4A-F), indicating no detectable influence on migration due to Rbm24 ablation. Furthermore, we explored the terminal development of adult-born neurons in the OB through virus labeling and electrophysiological recording. Similar spines density and AP firing rates of adult-born neurons were observed in the OB of UKO mice compared to CTL mice (Figure S5A-G), indicating that Rbm24 ablation does not affect the maturation of adult-born neurons in the OB. Overall, these results collectively suggest that Rbm24 deficiency impedes the adult neurogenesis in the OB.

### Rbm24 deficiency inhibits the proliferation of adult NSPCs from the SVZ *in vitro*

**Figure 4:**
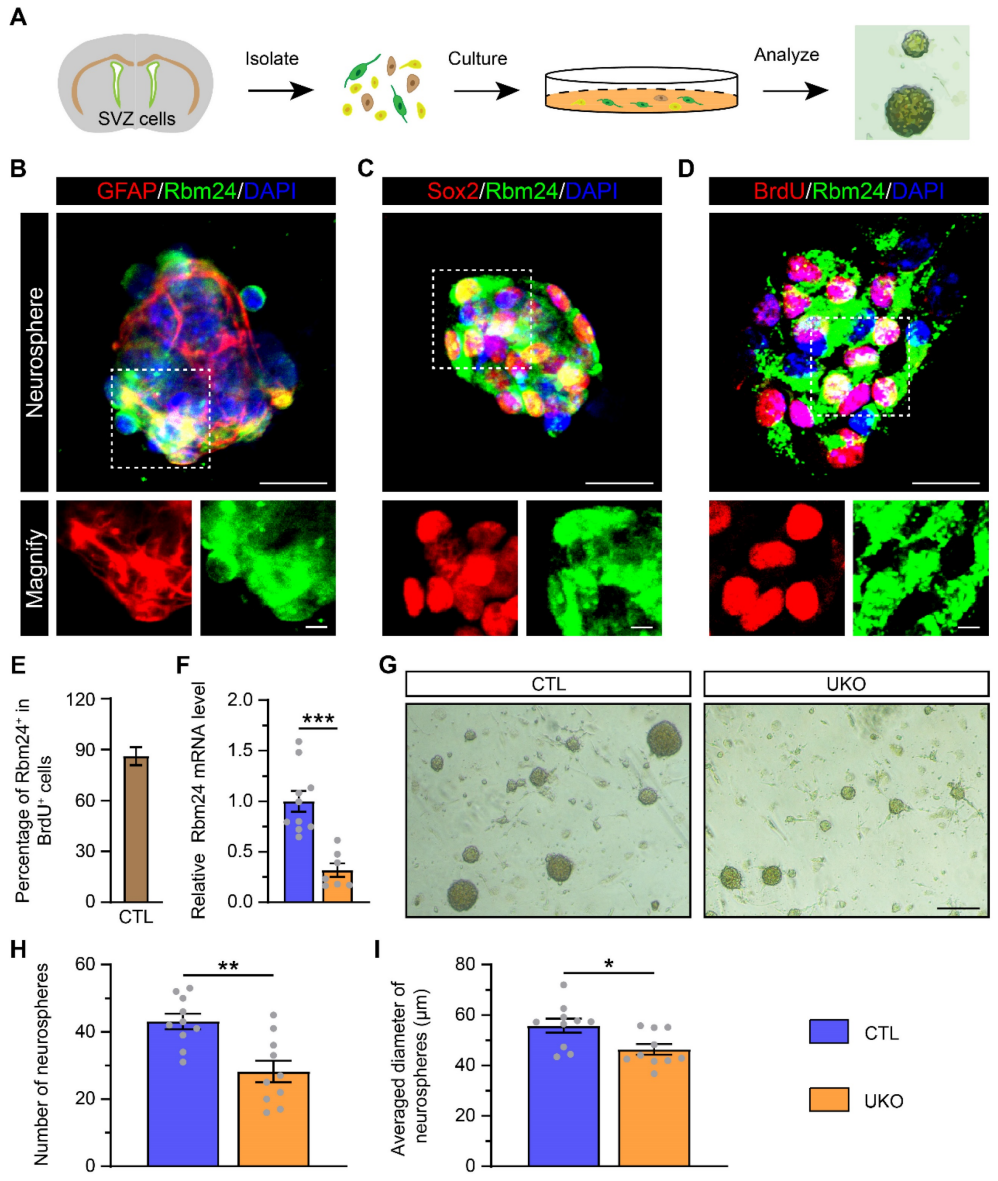
** Rbm24 deficiency inhibits the growth of neurosphere. (A)** Schematic diagram showing the primary culture of adult NSPCs derived from 2-month-old CTL and UKO mice. **(B-D)** Representative images of GFAP/Rbm24 (**B**), Sox2/Rbm24 (**C**), BrdU/Rbm24 (**D**) co-staining in the neurosphere from 2-month-old CTL mice. Scale bar, 20 μm, zoom scale bar, 5 μm. **(E)** Percentage of Rbm24^+^ in BrdU^+^ cells in (D) (n = 4 mice). **(F)** Quantification of Rbm24 mRNA in the neurosphere from 2-month-old CTL and UKO mice (n = 4 mice for each group). **(G)** Representative images of the neurosphere from 2-month-old CTL and UKO mice. Scale bar, 200 μm.** (H-I)** Quantification of number (**H**) and averaged diameter (**I**) of neurosphere in (**G**) from 2-month-old CTL and UKO mice (n = 5 mice for each group). Data are presented as mean ± SEM. *p < 0.05; **p < 0.01; ***p < 0.001.

Given the observed impact of Rbm24 deficiency on NSPC proliferation in the SVZ, we investigated its effects on cultured NSPCs. NSPCs were isolated from the SVZ of 2-month-old CTL mice and exhibited robust growth, forming neurospheres *in vitro* (Figure 4A). The identification of these neurospheres as NSPCs was determined by using GFAP and sex determining region Y-box 2 (Sox2) markers. Furthermore, both GFAP^+^ and Sox2^+^ cultured NSPCs demonstrated high expression of Rbm24, consistent with the *in-vivo* experiments (Figure 4B-C). Concurrently, BrdU incorporation revealed that the majority of cultured NSPCs were BrdU^+^/Rbm24^+^ cells, indicating an enrichment of Rbm24 in actively proliferating NSPCs (Figure 4D-E). Subsequent examination of cultured NSPCs derived from UKO mice revealed decreased Rbm24 mRNA levels relative to CTL mice (Figure 4F). A reduction in the size and number of neurospheres from UKO mice compared to CTL mice was also evident (Figure 4G-I). These findings collectively underscore the pivotal role of Rbm24 in the proliferation of adult NSPCs in the SVZ.

### Rbm24 deficiency changes the transcriptional profiles in the SVZ

**Figure 5:**
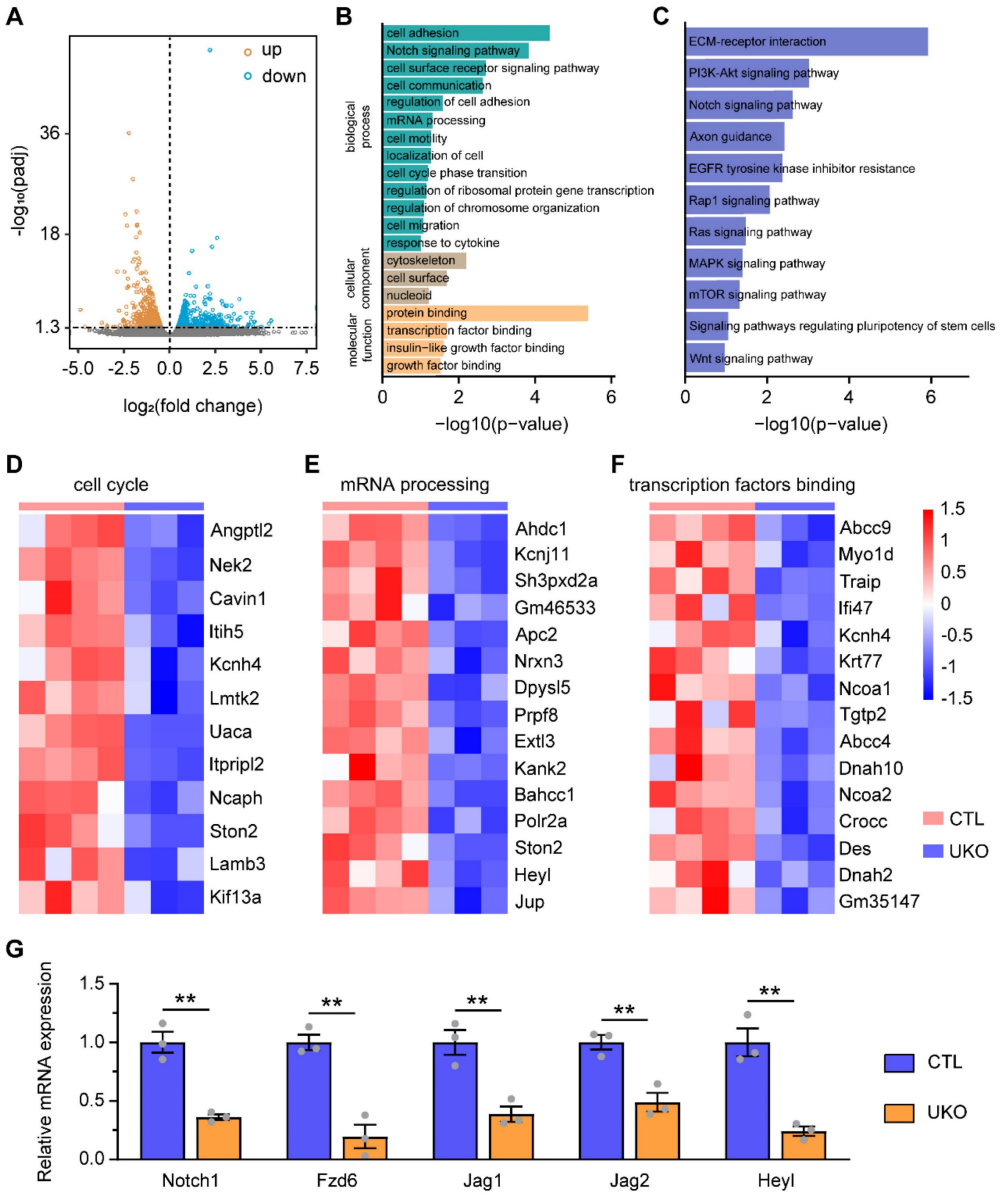
** Rbm24 deficiency changes the transcriptional profiles of the adult SVZ. (A)** Dot plot showing the DEGs in the SVZ between 2-month-old CTL and UKO mice in the RNA-Seq. **(B)** GO analysis of the down-regulated genes.** (C)** KEGG analysis of the down-regulated genes.** (D-F)** Heatmap illustrating the normalized expression of enriched cell cycle (**D**), mRNA processing (**E**), transcription factors binding (**F**) genes. **(G)** Quantification of indicated gene mRNA in the SVZ from 2-month-old CTL and UKO mice (n = 3 mice for each group). Data are presented as mean ± SEM. **p < 0.01.

To elucidate the molecular mechanisms underlying the role of Rbm24 in regulating adult neurogenesis, we conducted RNA-seq analysis on the SVZ derived from UKO and CTL mice. Compared to CTL mice, the RNA-seq analysis identified 1291 DEGs in UKO mice, consisting of 606 upregulated genes and 685 downregulated genes (Figure 5A). Subsequent GO analysis revealed that the downregulated DEGs were predominantly associated with various biological processes related to cell division, including the Notch signaling pathway, mRNA processing, and cell cycle phase transition (Figure 5B). Concurrently, KEGG analysis also highlighted several signaling pathways linked to cell proliferation, with a notable emphasis on the Notch signaling pathway (Figure 5C). Significantly, based on the RNA-seq analysis, Rbm24 deficiency led to a substantial downregulation of genes associated with proliferation and transcription (Figure 5D-F). Among the top 50 DEGs (Table S2), we postulated that the downregulated Notch1 might be a downstream target of Rbm24 in adult NSPCs in the SVZ, considering its similar expression pattern and known roles in SVZ neurogenesis 17. Subsequently, q-PCR analysis was performed to further validate the downregulation of Notch signaling pathway-related genes in the SVZ of UKO mice (Figure 5G). Considering the comparable effects of Notch1 and Rbm24 on adult neurogenesis in the SVZ, our subsequent investigations primarily focused on understanding the relationship between Rbm24 and Notch1.

### Rbm24 regulates the stability of Notch1 mRNA in adult NSPCs

**Figure 6:**
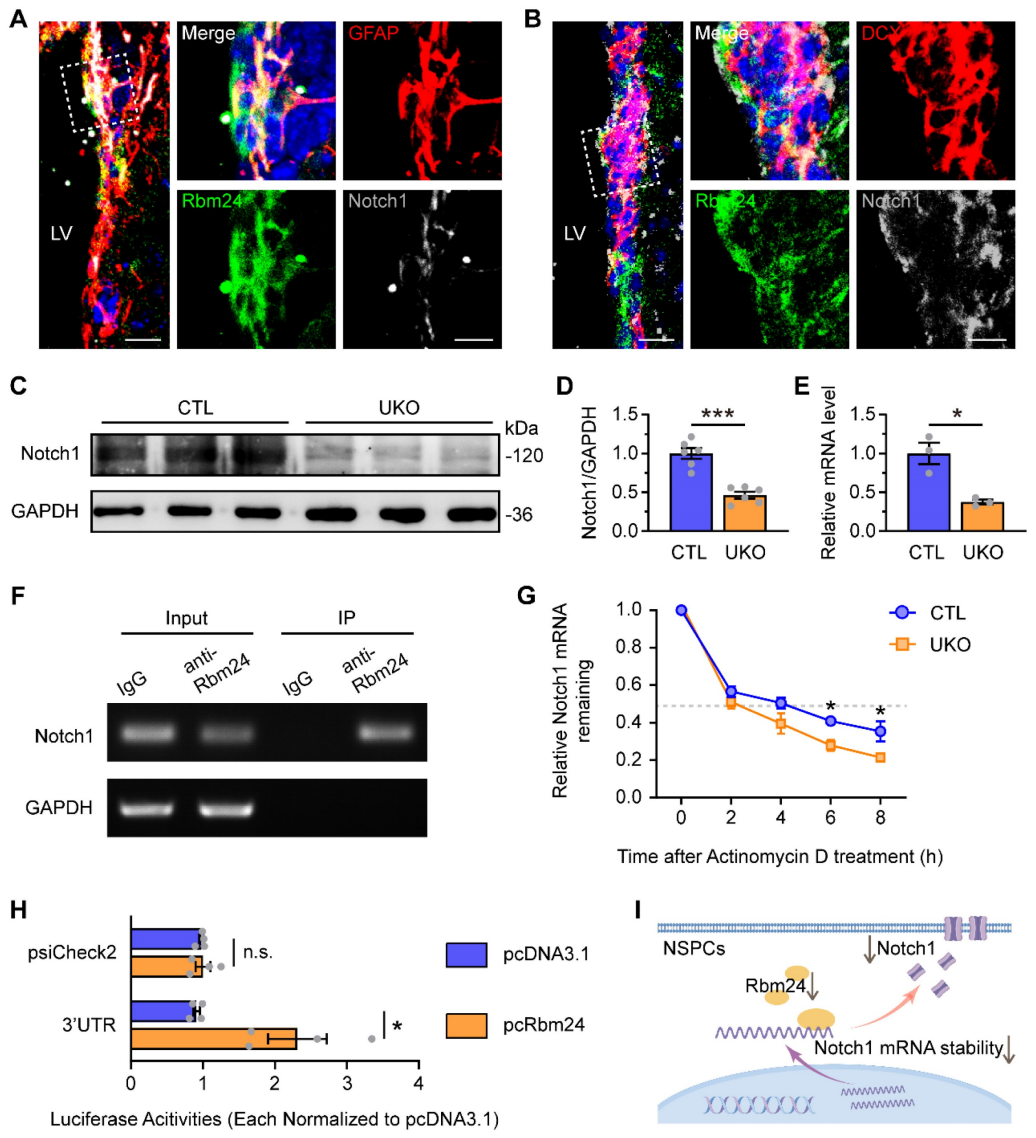
**Rbm24 directly regulates Notch1 mRNA stability in the adult NSPCs. (A-B)** Representative images of GFAP/Rbm24/Notch1 (**A**), DCX/Rbm24/Notch1 (**B**) co-staining in the SVZ from 2-month-old CTL mice. Scale bar, 20 μm, zoom scale bar, 10 μm. **(C-D)** Immunoblot (**C**) and quantification (**D**) of Notch1 protein in the SVZ from 2-month-old CTL and UKO mice (n = 6 mice for each group). **(E)** Quantification of Notch1 mRNA in the neurospheres from 2-month-old CTL and UKO mice (n = 3 mice for each group).** (F)** RT-PCR analysis of Notch1 mRNA in input and Rbm24-antibody IP in the SVZ from 2-month-old CTL mice. **(G)** Quantification of Notch1 mRNA in adult NSPCs from 2-month-old CTL and UKO mice with actinomycin D administration (n = 3 mice for each group). **(H)** Quantification of luciferase activities for 3'-UTR fragments of Notch1 in 293T cells between pcRbm24 and pcDNA3.1 condition (n = 4 for each group). **(I)** Schematic diagram showing the effects of Rbm24 deficiency on Notch1. Data are presented as mean ± SEM. *p < 0.05; ***p < 0.001; n.s., not significant. LV: lateral ventricle; NSPCs: neural stem/progenitor cells.

We initially confirmed the colocalization of Notch1 with Rbm24 in both GFAP^+^ and DCX^+^ NSPCs in the SVZ (Figure 6A-B). Following Rbm24 ablation, a significant reduction in Notch1 levels was observed in both the SVZ (Figure 6C-D) and cultured NSPCs (Figure 6E). To investigate whether Notch1 is a direct molecular target of Rbm24 in adult NSPCs, we conducted RNA immunoprecipitation (RNA-IP) using an Rbm24 antibody followed by PCR. The results demonstrated a direct binding of Rbm24 to Notch1 mRNA (Figure 6F). Furthermore, cultured NSPCs were treated with actinomycin D to inhibit transcription, and Notch1 mRNA levels were examined over an 8-h period in cultured NSPCs from UKO and CTL mice. The q-PCR analysis revealed a significant decrease in Notch1 mRNA levels at 6 and 8 h after actinomycin D treatment in cultured NSPCs from UKO mice, indicating a shortened half-life of Notch1 mRNA compared to NSPCs from CTL mice (Figure 6G). Further exploration was undertaken to identify the specific regions of Notch1 mRNA that interacted with Rbm24. Given that the RNA binding site of Rbm24 is a GT-rich region, we cloned the GT-rich region in the 3'-UTR regions of Notch1 mRNA into a pSicheck2 luciferase reporter vector, allowing the regulation of Renilla luciferase expression via the 3'-UTR sequence of Notch1 mRNA. When the reporter was co-transfected with Rbm24 expression plasmids (pcRbm24) into 293T cells, the overexpression of Rbm24 significantly increased luciferase activities compared to control vector (pcDNA3.1)-transfected conditions (Figure 6H). This result indicates an interaction of Rbm24 with the 3'-UTR sequence of Notch1 mRNA. Thus, these findings suggest that Rbm24 acts as a direct post-transcriptional mRNA stabilizer of Notch1 in adult NSPCs (Figure 6I), providing insights into the potential mechanisms by which Rbm24 regulates adult neurogenesis.

### Rbm24/Notch1 signaling axis is essential for adult neurogenesis of SVZ-OB pathway and olfaction in PD mice

**Figure 7:**
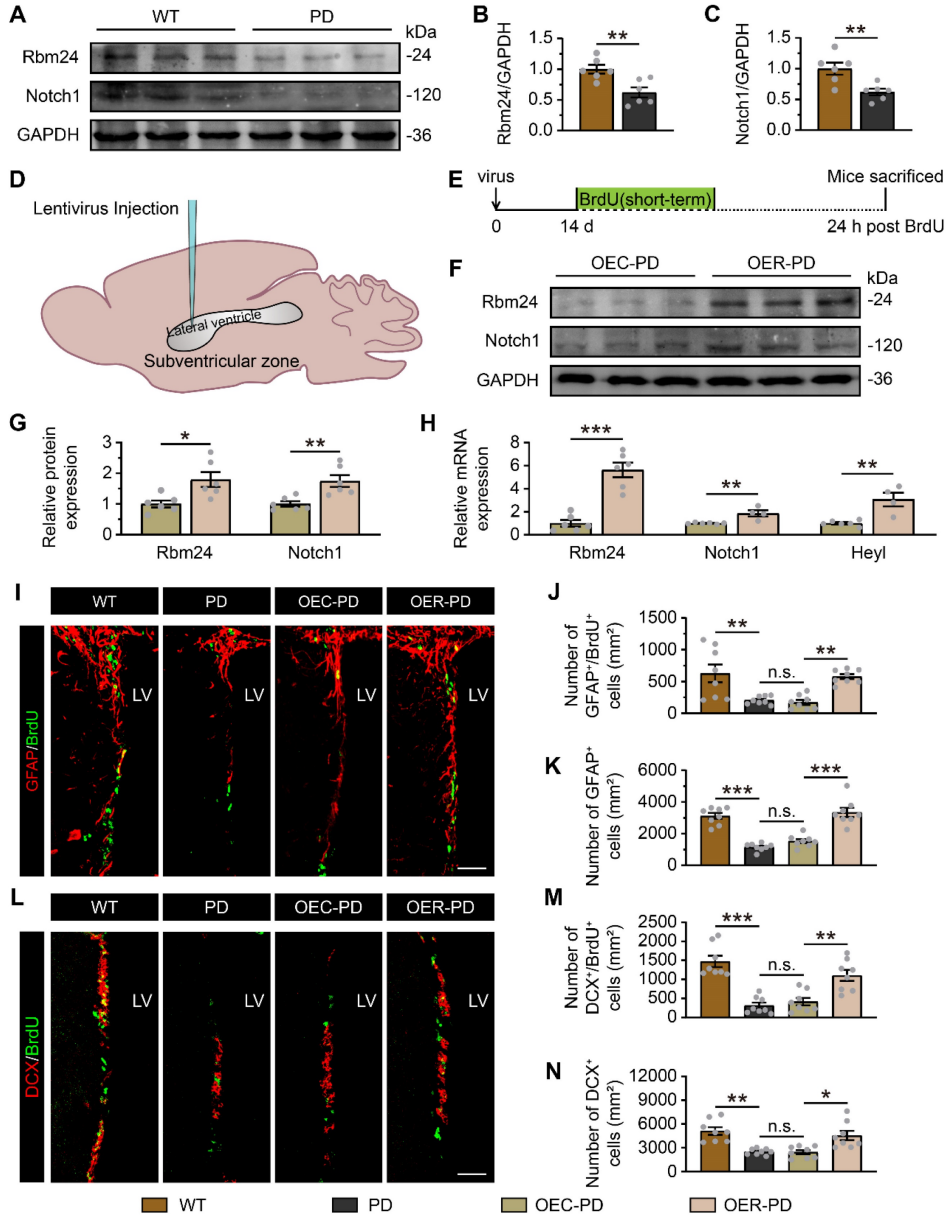
** Rbm24 overexpression ameliorates adult neurogenesis in the SVZ of PD mice. (A-C)** Immunoblot (**A**) and quantification of Rbm24 (**B**) and Notch1 (**C**) protein in the SVZ from 7-month-old WT and PD mice (n = 6 mice for each group). **(D)** Schematic diagram showing the lentivirus injection into the lateral ventricle.** (E)** Timeline of BrdU (short-term) and lentivirus injection in 7-month-old WT and PD mice. **(F-G)** Immunoblot (**F**) and quantification (**G**) of Rbm24 and Notch1 protein in the SVZ from 7-month-old OEC-PD and OER-PD mice (n = 6 mice for each group). **(H)** Quantification of Rbm24, Notch1, Heyl mRNA in the SVZ from 7-month-old OEC-PD and OER-PD mice (n = 6 mice for each group). **(I-K)** Representative images (**I**) and quantification of GFAP^+^/BrdU^+^ (**J**), GFAP^+^ (**K**) cells in the SVZ from 7-month-old WT, PD, OEC-PD, and OER-PD mice (n = 4 mice for each group). Scale bar, 50 μm. **(L-N)** Representative images (**L**) and quantification of DCX^+^/BrdU^+^ (**M**), DCX^+^ (**N**) cells in the SVZ from 7-month-old WT, PD, OEC-PD, and OER-PD mice (n = 4 mice for each group). Scale bar, 50 μm. Data are presented as mean ± SEM. *p < 0.05; **p < 0.01; ***p < 0.001; n.s., not significant. LV: lateral ventricle.

As mentioned earlier, the disruption of adult neurogenesis in the SVZ-OB pathway is a direct driver for olfactory dysfunction in neurodegenerative disease, particularly PD. The impact of Rbm24 ablation on SVZ neurogenesis exhibits Parkinson-like features. Therefore, we explored the potential role of Rbm24/Notch1 signaling axis in the adult neurogenesis of SVZ-OB pathway in a PD mouse model. Firstly, immunoblot analysis revealed a significant reduction in Rbm24 and Nocth1 protein levels in the SVZ of PD mice compared to wild-type (WT) mice (Figure 7A-C). To assess the effects of Rbm24 overexpression on SVZ neurogenesis in PD mice, we injected lentivirus encoding Rbm24-GFP (OER) or control-GFP (OEC) into the SVZ of PD mice (Figure 7D-E), confirmed by the immunoblot and q-PCR of Rbm24 (Figure 7F-H). Additionally, Rbm24 overexpression significantly upregulated the Notch1 and Heyl levels in the SVZ (Figure 7F-H).

**Figure 8:**
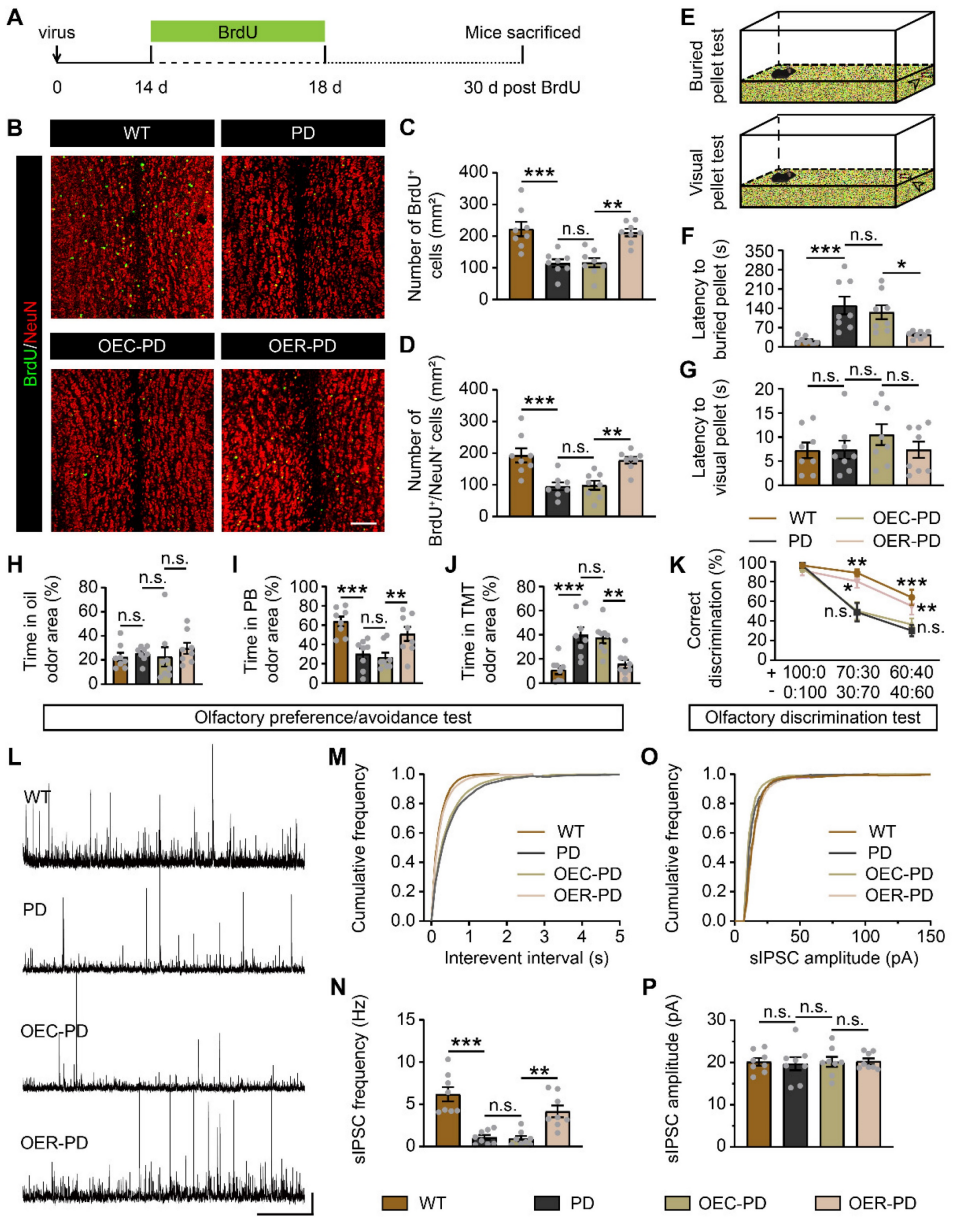
** Rbm24 overexpression improves OB neurogenesis and olfactory performance in PD mice. (A)** Timeline of BrdU and lentivirus injection in 7-month-old WT and PD mice. **(B-D)** Representative images (**B**) and quantification of BrdU^+^ (**C**), BrdU^+^/NeuN^+^ (**D**) cells in the OB from 8-month-old WT, PD, OEC-PD, and OER-PD mice (n = 4 mice for each group). Scale bar, 100 μm. **(E)** Schematic diagram illustrating the buried and visual pellet tests. **(F-G)** Quantification of latency to find a food pellet buried (**F**) or on the surface (**G**) of bedding in 9-month-old WT, PD, OEC-PD, and OER-PD mice (n = 8 mice for each group). **(H-J)** Percentage of time spend on exploring oil **(H)**, PB **(I)**, TMT **(J)** odor area for 9-month-old WT, PD, OEC-PD, and OER-PD mice (n = 8 mice for each group). (K) Percentage of correct olfactory discrimination per trial session in 9-month-old WT, PD, OEC-PD, and OER-PD mice (n = 8 mice for each group). **(L)** Representative traces of sIPSC of M/Ts in the OB from 9-month-old WT, PD, OEC-PD, and OER-PD mice. Scale bar, 40 pA and 20 s. **(M)** Cumulative curves of sIPSC interevent interval of M/Ts in (**L**). **(N)** Quantification of sIPSC frequency of M/Ts in (**L**) (n = 4 mice for each group). **(O)** Cumulative curves of sIPSC amplitude of M/Ts in (**L**). **(P)** Quantification of sIPSC amplitude of M/Ts in (**L**) (n = 4 mice for each group). Data are presented as mean ± SEM. *p < 0.05; **p < 0.01; ***p < 0.001; n.s., not significant.

Fewer BrdU^+^ cells, GFAP^+^/BrdU^+^ cells, GFAP^+^ cells, DCX^+^/BrdU^+^ cells, and DCX^+^ cells were observed in the SVZ of PD mice compared to WT mice, indicating impaired adult neurogenesis in the SVZ of PD mice (Figure 7I-N, Figure S6). However, Rbm24 overexpression increased the number of these cells in the SVZ of PD mice (Figure 7I-N, Figure S6). Furthermore, there were fewer BrdU^+^, BrdU^+^/NeuN^+^ cells in the OB of PD mice, which was rescued by Rbm24 overexpression (Figure 8A-D), suggesting a role for Rbm24 in the pathogenesis of PD.

We further examined whether Rbm24 could affect the olfactory performances of PD mice. In the buried/visual pellet tests, PD mice showed prolonged latency to locate buried food pellets, but not visual food pellets, compared to WT mice (Figure 8E-G), indicating impaired olfactory behaviors in PD mice. In the olfactory preference/avoidance test, PB served as a fond odor, while TMT represented the aversive odor, with mineral oil as the control. Remarkably, all groups showed similar exploration times for the filter paper containing mineral oil (Figure 8H). However, PD mice exhibited reduced exploration time for PB-scented filter paper relative to WT mice, while conversely spending more time investigating TMT-scented filter paper (Figure 8I-J). Additionally, in the olfactory discrimination test, mice were tasked with distinguishing different fractions of the mango and almond odor. The percentage of correct olfactory discrimination responses per trial in different fractions of smell mixtures was decreased in PD mice (Figure 8K). Notably, Rbm24 overexpression mitigated these olfactory deficits in PD mice, as indicated by improved odor preference and enhanced discrimination ability (Figure 8I-K). Meanwhile, electrophysiological experiments revealed a significant reduction in sIPSC frequency but unchanged amplitude of M/Ts in the OB of PD mice, which was rescued by Rbm24 overexpression (Figure 8L-P). Taken together, Rbm24 plays a critical role in the SVZ neurogenesis and olfaction in PD mice.

**Figure 9:**
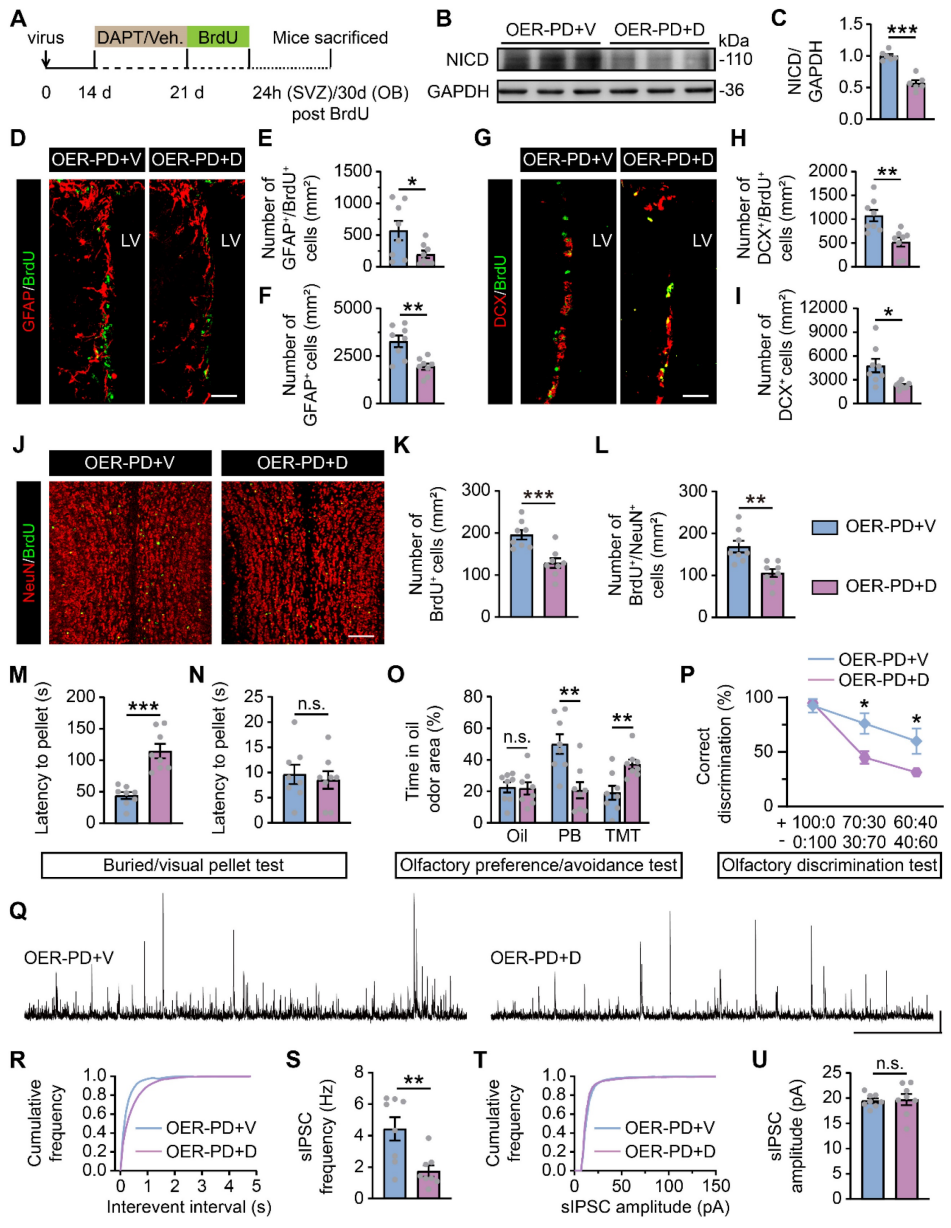
** Notch1 inhibition blocks the effects of Rbm24 overexpression on SVZ-OB pathway neurogenesis and olfactory performance in PD mice. (A)** Timeline of DAPT, BrdU and lentivirus injection in 7-month-old PD mice. **(B-C)** Immunoblot (**B**) and quantification (**C**) of NICD protein levels in the SVZ from 7-month-old OER-PD mice treated with vehicle or DAPT (n = 6 mice for each group). **(D-F)** Representative images (**D**) and quantification of GFAP^+^/BrdU^+^ (**E**), GFAP^+^ (**F**) cells in the SVZ from 7-month-old OER-PD mice treated with vehicle or DAPT (n = 4 mice for each group). Scale bar, 50 μm. **(G-I)** Representative images (**G**) and quantification of DCX^+^/BrdU^+^ (**H**), DCX^+^ (**I**) cells in the SVZ from 7-month-old OER-PD mice treated with vehicle or DAPT (n = 4 mice for each group). Scale bar, 50 μm. **(J-L)** Representative images (**J**) and quantification of BrdU^+^ (**K**), BrdU^+^/NeuN^+^ (**L**) cells in the OB from 8-month-old OER-PD mice treated with vehicle or DAPT (n = 4 mice for each group). Scale bar, 100 μm. **(M-N)** Quantification of latency to find a food pellet buried (**M**) or on the surface (**N**) of bedding in 9-month-old OER-PD mice treated with vehicle or DAPT (n = 8 mice for each group). **(O)** Percentage of time spend on exploring oil, PB, TMT odor area for 9-month-old OER-PD mice treated with vehicle or DAPT (n = 8 mice for each group). **(P)** Percentage of correct olfactory discrimination per trial session in 9-month-old OER-PD mice treated with vehicle or DAPT (n = 8 mice for each group). **(Q)** Representative traces of sIPSC of M/Ts in the OB from 9-month-old OER-PD mice treated with vehicle or DAPT. Scale bar, 40 pA and 20 s. **(R)** Cumulative curves of sIPSC interevent interval of M/Ts in (**Q**). **(S)** Quantification of sIPSC frequency of M/Ts in (**Q**) (n = 4 mice for each group). **(T)** Cumulative curves of sIPSC amplitude of M/Ts in (**Q**). **(U)** Quantification of sIPSC amplitude of M/Ts in (**Q**) (n = 4 mice for each group). Data are presented as mean ± SEM. *p < 0.05; **p < 0.01; ***p < 0.001; n.s., not significant. Veh.: vehicle; LV: lateral ventricle.

In parallel, DAPT, a potent Notch1 inhibitor, was administered i.p. to examine whether Notch1 is a downstream target of Rbm24 in adult SVZ neurogenesis (Figure 9A). Firstly, following DAPT treatment, a substantial decrease in Notch intracellular domain (NICD) was observed in the SVZ of PD mice with Rbm24 overexpression (Figure 9B-C), indicating an effective inhibition of Notch1 cleavage by DAPT treatment. Additionally, we observed that DAPT decreased the number of GFAP^+^/BrdU^+^ cells, GFAP^+^ cells, DCX^+^/BrdU^+^ cells, and DCX^+^ cells in the SVZ of PD mice with Rbm24 overexpression (Figure 9D-I). Furthermore, DAPT reduced the presence of BrdU^+^ and BrdU^+^/NeuN^+^ cells in the OB of PD mice overexpressing Rbm24 (Figure 9J-L). Additionally, the latency to locate buried pellets, not the visual pellets, was prolonged in PD mice with Rbm24 overexpression following DAPT administration (Figure 9M-N). Additionally, DAPT treatment decreased the duration spent investigating PB-scented filter paper and increased the duration spent sniffing TMT-scented filter paper in PD mice overexpressing Rbm24, while time spent exploring mineral oil-scented filter paper was similar in PD mice overexpressing Rbm24 with or without DAPT treatment (Figure 9O). In the olfactory discrimination test, DAPT treatment decreased the percentage of correct olfactory discrimination responses per trial in different fractions of smell mixtures in PD mice overexpressing Rbm24 (Figure 9P). Furthermore, DAPT treatment decreased sIPSC frequency of M/Ts in the OB of PD mice overexpressing Rbm24 (Figure 9Q-U), indicating that the effects of Rbm24 overexpression could be hindered by inhibiting Notch1. These findings suggest that Notch1 functions as a downstream target of Rbm24 in adult SVZ neurogenesis, playing an essential role in adult neurogenesis of the SVZ-OB pathway in PD mice.

## Discussion

Adult neurogenesis in the SVZ is crucial for maintaining olfaction, cognition and tissue restoration 41-44. Despite the direct involvement of NSPCs in the SVZ in developmental and neuropathological processes 19, 20, 45, the underlying mechanisms remain elusive. This study unveils a novel role of Rbm24 in adult neurogenesis in the SVZ under both physiological and pathological conditions. Our findings identify Notch1 as a direct downstream target of Rbm24, implicating the Rbm24/Notch1 signaling axis in Parkinson-associated olfactory dysfunction. These researches provide valuable insights into the mechanisms governing adult neurogenesis and suggest potential therapeutic targets for conditions associated with olfactory dysfunction and neurodegeneration.

Although Rbm24 has been extensively studied in the field of heart development, skeletal muscle regeneration and cancer 23-29, its roles in the central nervous system remain largely unexplored. This study revealed that Rbm24 was strongly expressed in neurogenic niches in embryonic and post-natal mice, particularly in the SVZ. Further confirmation showed the localization of Rbm24 in the GFAP^+^ and DCX^+^ NSPCs in the adult SVZ. *In-vivo* and *in-vitro* BrdU incorporation tests indicated that Rbm24 was predominantly present in actively proliferating NSPCs, with fewer Rbm24^+^ cells in quiescent NSCs, suggesting a potential role for Rbm24 in the overall development of NSPCs in the adult SVZ. Notably, Rbm24 distribution was restricted to the SVZ, with scarce presence in the RMS and OB during NSPC migration, hinting at a potential initiation role in adult neurogenesis in the SVZ-OB pathway.

The NSPC pool in the SVZ undergoes dynamic changes and serves as the source of adult-born neurons in the central nervous system 46, 47. Although previous study has shown that Rbm24 could regulate cellular development in multiple organs 32, its specific involvement in the dynamic regulation of NSPCs remains unclear. This study revealed that Rbm24 ablation significantly inhibited NSPC proliferation, particularly in the actively proliferating NSPCs, with minimal impact on the quiescent ones. This discrepancy may arise from the potential distinct regulatory processes in quiescent and activated NSPCs. Additionally, the inhibitory effects on NSPC proliferation due to Rbm24 depletion were consistently observed in cultured adult NSPCs. Of note, the progeny cells of NSPCs in the SVZ typically migrate and mature in the OB, giving rise to various interneurons, including TH^+^, CalR^+^ and CalB^+^ cells 6. Our results demonstrated a decrease in the number of these interneurons 60 days after TAM injection, accompanied by a defective inhibitory effect of M/Ts in the OB. However, evaluating whether the disruption of adult neurogenesis by Rbm24 depletion leads to impaired olfactory behavior proves challenging due to the motor coordination defect observed in UKO mice 26. Nevertheless, previous studies suggest that a weak inhibitory neuronal microcircuit restricted in the OB alone can induce olfactory dysfunction in mice 48, 49. Hence, we hypothesize that Rbm24 ablation could result in aberrant olfactory performance in mice.

As an evolutionarily conserved RBP, Rbm24 is recognized for its role in regulating alternative splicing and mRNA stability 23, 25-27, 30. In an attempt to elucidate the regulatory mechanisms, we conducted an RNA-seq analysis, identifying Notch1 as a potential target of Rbm24 among the top 50 DEGs. As previously reported 50, 51, Notch1 is located in the mitotic and mitotically inactive NSPCs in the SVZ, primarily in DCX^+^ neuroblasts. Additionally, Notch1 exhibits a gradual decrease along the SVZ-OB pathway, with a lesser extent in cells in the OB and olfactory cortex 52. These findings indicate that Notch1 is particularly enriched in the initiation of the SVZ-OB pathway, a distribution comparable to that of Rbm24. Moreover, mounting studies have demonstrated that Notch1 ablation or inhibition can lead to the loss of active NSPCs in SVZ homeostasis and result in a loss of both quiescent and active NSPCs in SVZ regeneration 17, 53, 54, similar to the effects observed with Rbm24 deletion. Thus, the analogous distribution and functions of Rbm24 and Notch1 raise the possibility of an interaction between them. Subsequent binding assays confirmed a direct interaction between Rbm24 and Notch1 mRNA, with Rbm24 binding to the 3'-UTR of Notch1 mRNA to maintain stability. While this study primarily focuses on the interaction between Rbm24 and Notch1, the potential involvement of other signaling factors in the impaired SVZ neurogenesis induced by Rbm24 ablation, such as PI3K/Akt, mTOR, and Wnt signaling 55-58, requires additional exploration in the future.

Compelling evidence has demonstrated that the disruption of adult neurogenesis in the SVZ is implicated in non-motor symptoms in the early stages of PD, significantly contributing to the disease progression 10, 59-62. Specifically, the human A30P point mutation in the *SNCA* gene has been shown to induce a loss of actively proliferating NSPCs in the SVZ, along with a reduction in interneurons in the OB 61. In this study, a decrease in both active and quiescent NSPCs in the SVZ, similar defective OB neurogenesis, and impaired olfactory behaviors were observed in the specific PD model mice carrying human A53T point mutation in the *SNCA* gene. Furthermore, emerging evidence suggests that gangliosides can potentially restore impaired neurogenesis and decelerate disease progression in the A53T PD model mice 9. However, the precise targets for reversing the loss of NSPCs and adult-born neurons remain significantly elusive.

Considering that the phenotypes of Rbm24 deletion exhibit Parkinson-like features in the SVZ-OB pathway, this study further explored the relevance of Rbm24/Notch1 signaling in PD. Intriguingly, downregulated Rbm24/Notch1 signaling was observed in the SVZ of PD mice. Additionally, lentiviral-mediated Rbm24 overexpression in the SVZ of PD mice enhanced NSPC proliferation, improved olfactory behaviors, and rescued electrophysiological deficits. Meanwhile, the effects of Rbm24 overexpression were hindered by the Notch1 inhibitor DAPT, suggesting Notch1 as a downstream target of Rbm24 in SVZ neurogenesis during PD pathogenesis. Furthermore, Rbm24 overexpression increased the total amount of GFAP^+^ NSCs, aligning with the roles of Notch1 53, 54, but differing from the physiological role of Rbm24. Therefore, Rbm24 appears to play distinct roles in both physiology and pathology. This study concludes that targeting Rbm24/Notch1 signaling holds therapeutic potential in PD. However, the cause of dysregulated Rbm24/Notch1 signaling in PD mice remains to be studied. Several studies have identified that α-synuclein deposits in the SVZ of PD mouse models are almost undetectable, but they are evident in the striatum adjacent to the SVZ, potentially affecting NSPC dynamic regulation through secretory substances, such as vulnerable dopamine 59, 61, 63. This prompts further investigation into whether the aberrant SVZ neurogenesis is a secondary effect in α-synuclein overexpressed mice.

Furthermore, another neurogenic niches in the mammalian brain, the DG, also host postnatal NSPCs. NSPCs in the DG share a similar transcriptional regulatory state with those in the SVZ. These DG-resident NSPCs give rise to new neurons and astrocytes, replenishing their initial population. Newly formed granule cells and astrocytes can integrate into the local circuits and promote hippocampus-related functions, particularly cognition. However, PD patients and models with cognitive dysfunction often exhibit alterations in several neurogenic cell populations in the DG, accompanied by maturation impairments 62, 64, 65. Additionally, adult neurogenesis in the DG of PD mice is regulated by both intracellular and extracellular molecular, such as Wnt or Notch signaling pathways and microglia-related neuroinflammation 66, 67. Considering the effects of the Rbm24/Notch1 axis on the SVZ neurogenesis, it is plausible to speculate that Rbm24 might be involved in maintaining adult neurogenesis in the DG, although this remains to be identified in future studies.

## Conclusions

In summary, our study highlights the essential role of Rbm24 in regulating the activity of dynamic NSPC populations, mediated through Notch1. The Rbm24/Notch1 signaling axis plays a crucial role in maintaining adult neurogenesis in both homeostasis and pathological regeneration. The study concludes that targeting the Rbm24/Notch1 signaling axis could be a promising strategy for the treatment of early-stage PD and related neurological disorders.

## Supplementary Material

Supplementary figures and tables.

## Abbreviations

SVZ: subventricular zone
LV: lateral ventricles
SGZ: subgranular zone
DG: dentate gyrus
NSPCs: Neural stem/progenitor cells
OB: olfactory bulb
PD: Parkinson's disease
RBPs: RNA binding proteins
Rbm24: RNA binding motif protein 24
TAM: tamoxifen
RNA-Seq: RNA-sequencing
NSCs: neural stem cells
BrdU: 5-bromo-2'-deoxyuridine
CTL: Control
UKO: Inducible knockout
i.p.: intraperitoneally
RMS: rostral migratory stream
DAPT: N-[N-(3,5-difluorophenacetyl)-l-alanyl]-Sphenylglycinet- butyl ester
AAV1: adeno-associated virus 1
EGFP: Enhanced green fluorescent protein
CMV: Cytomegalovirus
GFP: Green fluorescent protein
PBS: phosphate buffered saline
PFA: paraformaldehyde
BSA: bovine serum albumin
q-PCR: quantitative PCR
RIPA: Radioimmunoprecipitation Assay
DEGs: differentially expressed genes
FDR: false discovery rate
GO: Gene Ontology
KEGG: Kyoto Encyclopedia of Genes and Genomes
AP: action potential
sIPSC: spontaneous inhibitory postsynaptic current
M/Ts: mitral/tufted cells
PB: peanut butter
TMT: 2,4,5-trimethylthiazoline
Mgo: mango extract
ALM: almond extract
DB: denatonium benzoate
GFAP^+^: glial fibrillary acidic protein-positive
DCX^+^: doublecortin-positive
CalR^+^: calretinin-positive
CalB^+^: calbindin-positive
TH^+^: tyrosine hydroxylase-positive
Sox2: Sex determining region Y-box 2
RNA-IP: RNA immunoprecipitation
WT: wild-type
OER: lentivirus encoding Rbm24-GFP
OEC: lentivirus encoding control-GFP
NICD: Notch intracellular domain
