# Supplementary material for: Rbm24/Notch1 signaling regulates adult neurogenesis in the subventricular zone and mediates Parkinson-associated olfactory dysfunction
Source: Theranostics. 2024 Aug 1;14(11):4499–518. doi: 10.7150/thno.96045 (PMC11303084; doi:10.7150/thno.96045)
Supplement: Supplementary file 1 — Supplementary figures and tables. [file thnov14p4499s1.pdf]

Supplemental material

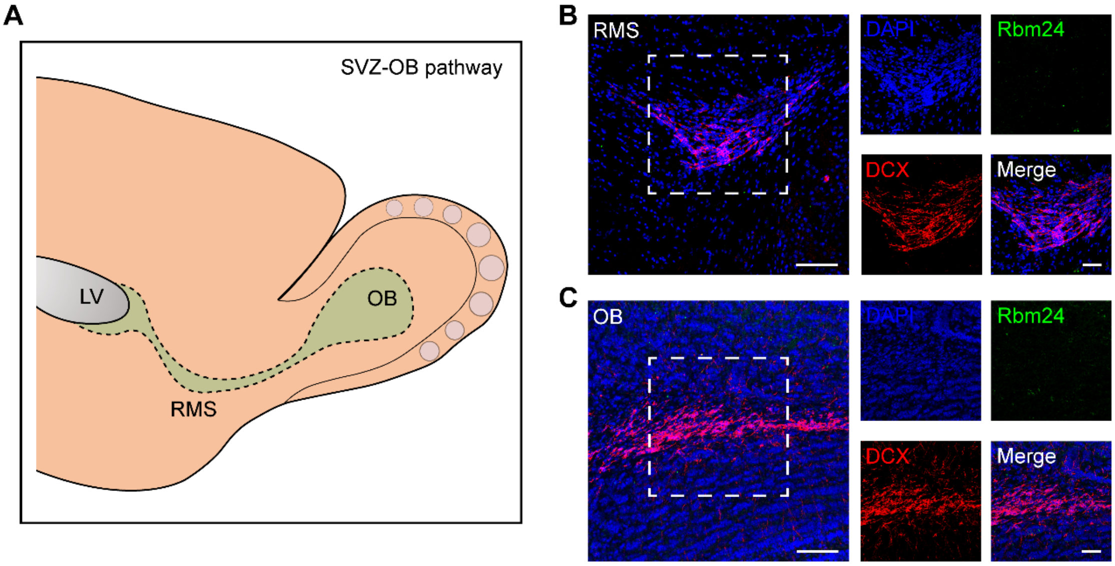

Figure S1. Rbm24 is rare in the neuroblasts of RMS and OB. (A) Schematic diagram illustrating the SVZ-OB pathway. **(B-C)** Representative images of Rbm24/DCX co-staining in the RMS (**B**) and OB (**C**) from 2-month-old CTL mice. Scale bar, 100 µm, zoom scale bar, 50 µm. LV: lateral ventricle; RMS: rostral migratory steam; OB: olfactory bulb.

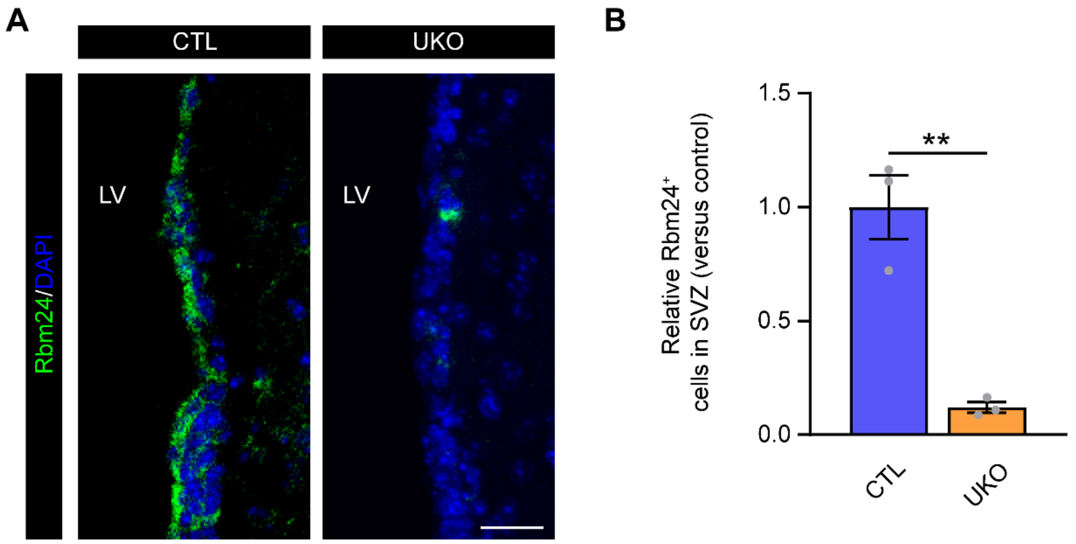

Figure S2. Rbm24⁺ cells are significantly reduced in the SVZ of UKO mice. (A-B) Representative images (**A**) and quantification (**B**) of Rbm24⁺ cells in the SVZ from 2-month-old CTL and UKO mice (n = 3 mice for each group). Scale bar, 25 μm. Data are presented as mean ± SEM. **p < 0.01. LV: lateral ventricle.

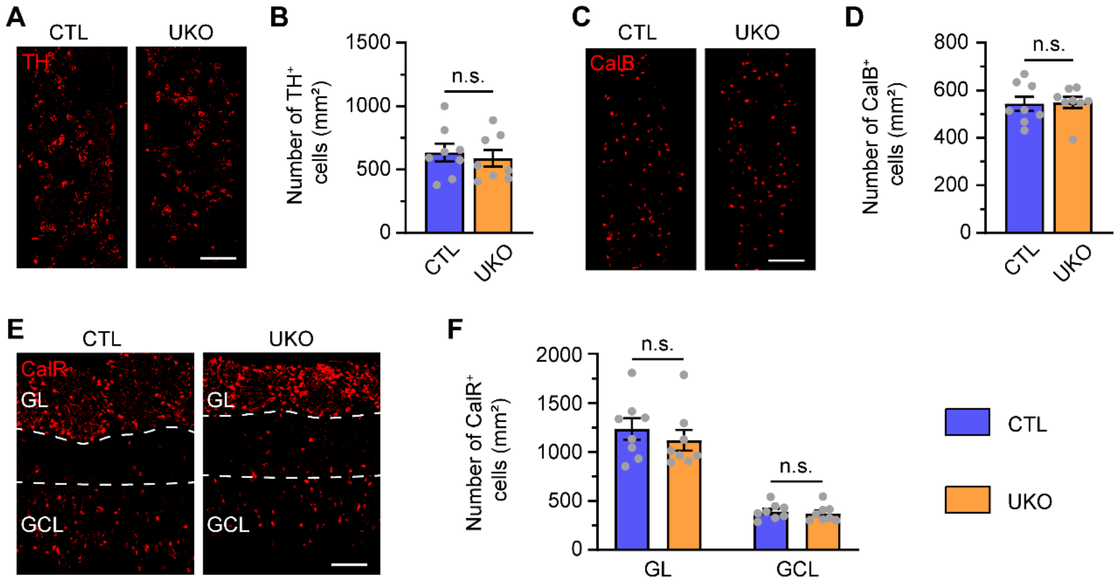

Figure S3. Rbm24 ablation does not significantly affect the amounts of interneurons in the OB 40 days after TAM injection. (A-F) Representative images and quantification of TH$^+$ (**A, B**), CalB$^+$ (**C, D**), CalR$^+$ (**E, F**) cells in the OB from 3-month-old CTL and UKO mice 40 days after tamoxifen administration (n = 4 mice for each group). Scale bar, 100 µm. Data are presented as mean ± SEM. not significant. GL: glomerular layer; GCL: granule cell layer.

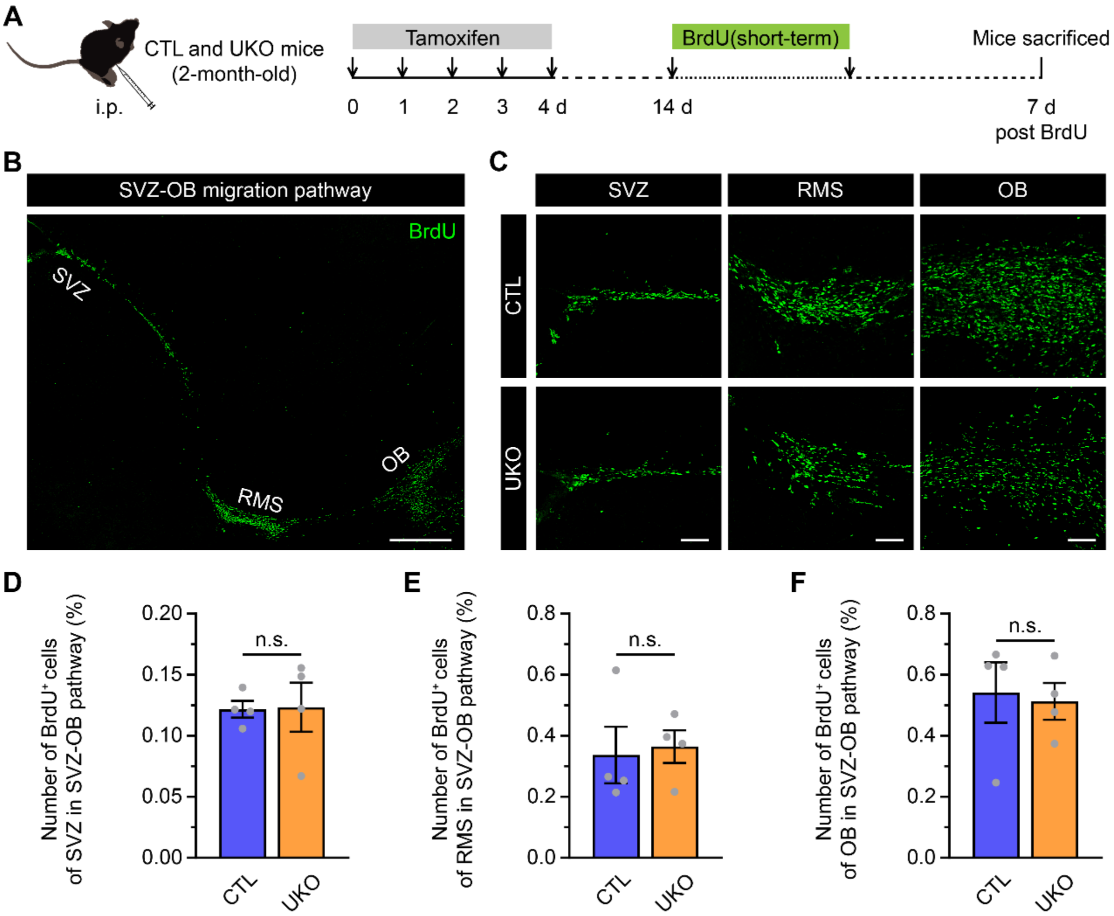

Figure S4. Rbm24 ablation does not significantly affect the migration of neuroblasts in the SVZ-OB pathway. (A) Timeline of tamoxifen and BrdU (short-term) administration in 2-month-old CTL and UKO mice. **(B)** Representative images of BrdU^{+} cells in the SVZ-OB pathway from 2-month-old CTL mice. Scale bar, 500 μm. **(C)** Representative images of BrdU^{+} cells in the SVZ, RMS, OB from 2-month-old CTL and UKO mice. Scale bar, 100 μm. **(D-F)** Percentage of BrdU^{+} cells in the SVZ (**D**), RMS (**E**), OB (**F**) from 2-month-old CTL and UKO mice (n = 3 mice for each group). Data are presented as mean ± SEM. n.s., not significant. i.p.: intraperitoneally; SVZ: subventricular zone; RMS: rostral migratory steam; OB: olfactory bulb.

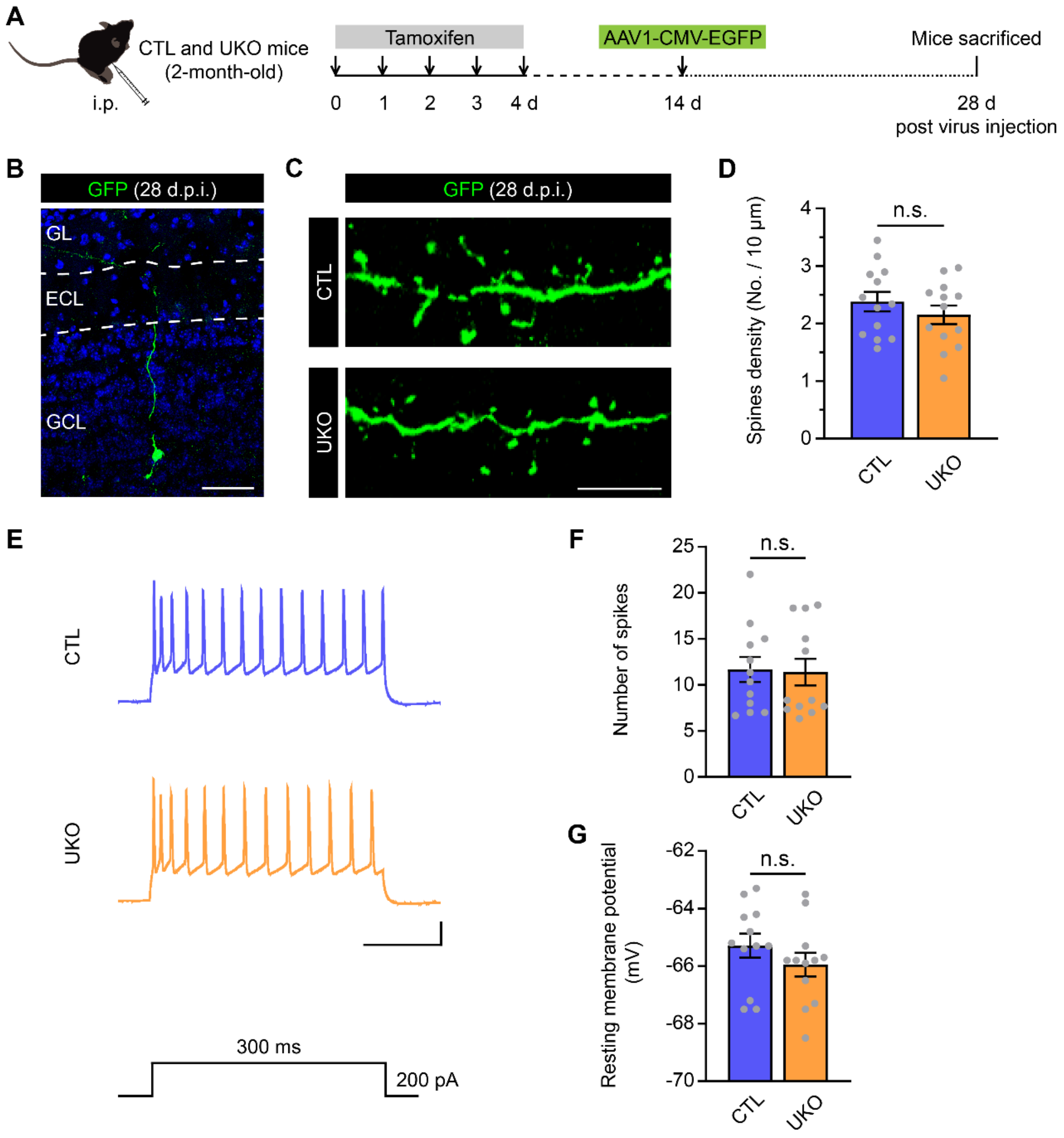

Figure S5. Rbm24 ablation does not significantly affect the terminal development of adult-born neurons in the OB. (A) Timeline of tamoxifen and AAV1-CMV-EGFP injection in 2-month-old CTL and UKO mice. **(B)** Representative images of virus-labeling cells in the OB from 3-month-old CTL mice. Scale bar, 50 μm. **(C-D)** Representative images (**C**) and quantification (**D**) of spines density of virus-labeling cells in the OB from 3-month-old CTL and UKO mice (n = 4 mice for each group). Scale bar, 10 μm. **(E)** Representative traces of action potential of virus-labeling cells under the stimulus (300 ms, 200 pA) in the OB from 3-month-old CTL and UKO mice. Scale bar, 20 mV and 100 ms. **(F-G)** Quantification of spikes number (**F**) and resting membrane potential (**G**) of virus-labeling cells in the OB from 3-month-old CTL and

UKO mice (n = 4 mice for each group). Data are presented as mean ± SEM. n.s., not significant. i.p.: intraperitoneally; d.p.i.: days post injection; GL: glomerular layer; ECL: external plexiform layer; GCL: granule cell layer.

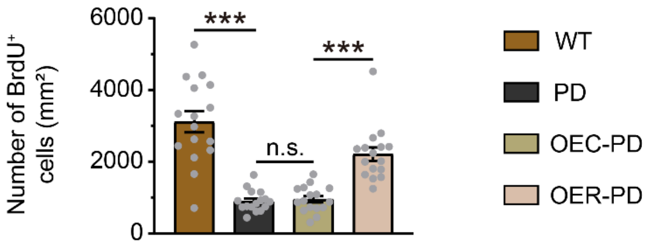

Figure S6. Rbm24 overexpression promotes cell proliferation in the SVZ of PD mice (related to Figure 7). Quantification of $BrdU^+$ cells in the SVZ from 7-month-old WT, PD, OEC-PD, and OER-PD mice (n = 4 mice for each group). Data are presented as mean ± SEM. ***$p < 0.001$; n.s., not significant.

Table S1. PCR primers

Gene	Forward	Revers
Rbm24	CCAAGGATCATGCAACCAG	GCAGGTATCCCGAAAGGTCT
Notch1	CAGACCAACACGCAGTACCA	AGAGTGACGTCAATGCC
Fzd6	AGACAACATTAGCGGCGTTT	AGAGGAGAGACAGCCCAACA
Jag1	AGCTTCGGCTCAGGGTCTA	TGCTATCAGGTTGAATAGTGTCA
Jag2	CCTGTGTGGTTATCTGCGTAT	GCTCTCATCCCGTGGTAG
Heyl	TGGAGAAAGCCGAGGTCTTGC	ACCTGATGACCTCAGTGAGGCA

Table S2. Top 50 DEGs identified by RNA-seq in the SVZ between CTL and UKO mice

Gene symbol	Readcount (UKO)	Readcount (CTL)	log2FoldChange	*P*-value	*P*-adj
gene-Xist	12.11037	16904	-10.447	0	0
gene-Malat1	50761.64	10977.37	2.2092	1.66E-55	8.34E-52
gene-Flt1	307.332	1437.105	-2.2253	1.67E-40	7.20E-37
gene-Fn1	267.8995	1080.062	-2.0114	3.00E-32	1.13E-28
gene-Adgrf5	276.9148	966.3372	-1.8031	2.40E-26	8.05E-23
gene-Xdh	52.37065	281.0038	-2.4238	9.19E-26	2.77E-22
gene-Abcb1a	215.025	736.9288	-1.777	6.33E-24	1.74E-20
gene-Podxl	192.1301	670.3348	-1.8028	1.05E-23	2.63E-20
gene-Kdr	76.01412	380.8344	-2.3248	1.22E-23	2.83E-20
gene-Ptprb	291.1416	911.8322	-1.647	2.17E-22	4.68E-19
gene-Adgrl4	130.0234	465.2437	-1.8392	1.41E-21	2.84E-18
gene-Gm32262	161.7858	26.55595	2.607	2.32E-21	4.37E-18
gene-LOC115488769	182.2774	36.3832	2.3248	9.25E-20	1.64E-16
gene-mt-Rnr1	104187.7	44587.07	1.2245	5.32E-19	8.90E-16
gene-Trhr2	96.14431	340.0212	-1.8224	1.10E-18	1.75E-15
gene-Eng	111.0842	365.0313	-1.7164	1.30E-17	1.96E-14
gene-Cdh5	65.47783	249.9788	-1.9327	2.15E-17	3.09E-14
gene-Myh11	53.39125	213.5129	-1.9996	6.00E-17	8.22E-14
gene-Vwf	115.8422	367.7976	-1.6668	8.06E-17	1.06E-13
gene-Hspg2	99.23888	325.1537	-1.7121	2.08E-16	2.62E-13
gene-Slc22a8	70.53939	246.1192	-1.8029	1.15E-15	1.39E-12
gene-Ackr2	12.28769	89.06646	-2.8577	3.77E-15	4.37E-12
gene-Flt4	22.42193	125.4986	-2.4847	4.33E-15	4.83E-12
gene-Slc7a5	386.5535	940.5506	-1.2828	5.91E-15	6.37E-12
gene-Pcdhga7	144.297	408.3277	-1.5007	7.88E-15	8.19E-12
gene-mt-Rnr2	123599.3	59094.15	1.0646	9.02E-15	9.06E-12

gene-Igsf9	106.185	320.4861	-1.5937	9.41E-15	9.15E-12
gene-Cgnl1	155.416	413.5836	-1.412	1.00E-13	9.46E-11
gene-Sema3g	23.95312	116.2811	-2.2793	1.05E-13	9.55E-11
gene-Tns1	645.1202	1483.753	-1.2016	2.96E-13	2.63E-10
gene-Stra6	55.99486	188.8159	-1.7536	5.30E-13	4.56E-10
gene-Robo4	35.31151	136.0263	-1.9457	1.80E-12	1.51E-09
gene-D830031N03Rik	118.895	27.67256	2.1032	2.40E-12	1.96E-09
gene-Mmrn2	21.74113	104.2367	-2.2614	2.82E-12	2.18E-09
gene-Tie1	55.20314	192.4321	-1.8015	2.81E-12	2.18E-09
gene-Apex2	276.0672	101.9897	1.4366	3.15E-12	2.38E-09
gene-Ptprm	548.2922	1180.138	-1.1059	3.75E-12	2.76E-09
gene-She	18.32846	92.53312	-2.3359	4.97E-12	3.57E-09
gene-Slco1a4	486.3744	1040.437	-1.0971	6.57E-12	4.60E-09
gene-Nos3	75.3977	220.9572	-1.5512	1.06E-11	7.25E-09
gene-Notch1	451.23	970.7236	-1.1052	1.33E-11	8.90E-09
gene-Cxcl12	492.4512	1095.717	-1.1538	1.61E-11	1.03E-08
gene-Pcdhga9	156.5681	383.7403	-1.2933	1.59E-11	1.03E-08
gene-Clic5	50.43314	162.1085	-1.6845	3.26E-11	2.00E-08
gene-Sox17	37.81766	133.3704	-1.8183	3.22E-11	2.00E-08
gene-Bahcc1	408.5002	866.9432	-1.0856	4.15E-11	2.50E-08
gene-Rgs5	1123.469	2231.467	-0.99003	4.46E-11	2.63E-08
gene-Slc2a1	748.1605	1522.78	-1.0253	4.96E-11	2.88E-08
gene-Foxo1	1630.935	3343.365	-1.0356	5.72E-11	3.25E-08
gene-Svil	323.6247	697.8225	-1.1085	6.12E-11	3.42E-08
